# A Selective Deposition Strategy of Ultrathin Metal Layer on Sub‐Micrometer‐Pitch Cu Interconnection for Low‐Temperature Hybrid Bonding

**DOI:** 10.1002/smsc.202500271

**Published:** 2025-10-11

**Authors:** Zambaga Otgonbayar, Jungchul Noh, Seong‐Ho Yoon, Gyu‐Sik Park, Suk Jekal, Jiwon Kim, Jeong‐Hwan Lee, Rino Choi, Jeonghun Kim, Myeongjin Kim, Chang‐Min Yoon

**Affiliations:** ^1^ Department of Polymer Science and Engineering Inha University 100 Inha‐ro, Michuhol‐gu Incheon 22212 Korea; ^2^ Department of Chemical Engineering Hongik University 94 Wausan‐ro, Mapo‐gu Seoul 04066 Korea; ^3^ Interdisciplinary Graduate School of Engineering Sciences Kyushu University 6‐1 Kasugakoen, Kasuga Fukuoka 816‐8580 Japan; ^4^ Institute for Materials Chemistry and Engineering Kyushu University 6‐1 Kasugakoen, Kasuga Fukuoka 816‐8580 Japan; ^5^ Program in Environmental and Polymer Engineering Inha University 100 Inha‐ro, Michuhol‐gu Incheon 22212 Korea; ^6^ Department of Materials Science and Engineering Inha University 100 Inha‐ro, Michuhol‐gu Incheon 22212 Korea; ^7^ Program in Semiconductor Convergence Inha University 100 Inha‐ro, Michuhol‐gu Incheon 22212 Korea; ^8^ 3D Convergence Center Inha University 100 Inha‐ro, Michuhol‐gu Incheon 22212 Korea; ^9^ Department of Chemical and Biomolecular Engineering Yonsei University 50 Yonsei‐ro, Seodaemun‐gu Seoul 03722 Korea; ^10^ Department of Hydrogen & Renewable Energy Kyungpook National University 80 Daehak‐ro, Bukgu Daegu 41566 Korea

**Keywords:** advanced packaging, Cu—Cu metallic bonds, electroless deposition, hybrid bonding, low‐temperature bonding

## Abstract

The advancement of high‐performance, miniaturized electronic systems continues to drive the development of advanced semiconductor packaging technologies with high bandwidth and integration density. Among these, sub‐micrometer‐pitch Cu interconnection via Cu/SiO_2_ hybrid bonding has become a critical technique for achieving vertical chip stacking in 3D integration. However, Cu—Cu direct bonding often requires process temperatures exceeding 400 °C to facilitate the metal diffusion, which poses challenges in back‐end‐of‐line compatibility and thermal stress management. In this study, a thermally efficient hybrid bonding method based on electroless deposition (ELD) of metal is presented, enabling interconnect formation at significantly lower temperatures. Ultrathin metal interlayers of gold (Au), platinum (Pt), and tin (Sn) (10–40 nm) are selectively deposited on 500 nm Cu pads using a solution‐based process, ensuring no impact on the adjacent SiO_2_ dielectric layer. The bonding process involves vacuum pre‐bonding at 120 °C, followed by annealing at 250 °C, achieving strong Cu—Cu diffusion and void‐free interfacial formation. This selective ELD‐assisted bonding method provides control over interface chemistry and ensures robust dielectric bonding through complementary surface silylation. The proposed process offers a scalable, low‐temperature pathway for fine‐pitch hybrid bonding, providing a promising solution for next‐generation ultradense 3D integrated semiconductor packaging.

## Graphical abstract

Conventional hybrid bonding approaches require high temperatures and long annealing times, limiting their compatibility with advanced semiconductor packaging. To overcome this limitation, various interfacial metals have been selected to enable uniform electroless deposition on sub‐micrometer Cu pads while preserving dielectric integrity, offering a low‐temperature pathway for next‐generation 3D packaging.

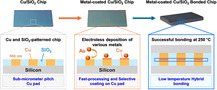

## 1 Introduction

The rapid progression of semiconductor technology continues to be fueled by the growing demands of diverse applications such as autonomous vehicles, artificial intelligence systems, and high‐performance computing.^[^ ^1^, ^2^, ^3^ ^]^ These emerging domains require interconnect solutions that support high‐speed data transmission, reduced power consumption, and compact system integration. As such, the development of high‐density semiconductor packaging technologies has become essential for enabling next‐generation system architectures.^[^ ^4^ ^]^ Although conventional interconnect approaches, such as solder bumping,^[^ ^5^, ^6^ ^]^ thermocompression bonding,^[^ ^7^ ^]^ and microbump techniques,^[^ ^8^ ^]^ have long provided reliable solutions, they are increasingly constrained by limitations in pitch scalability, thermal management, and process complexity. In response to these challenges, hybrid bonding, which enables direct metal–metal and dielectric–dielectric contact, has emerged as a promising technology for achieving highly integrated, high‐performance, and bandwidth‐rich semiconductor packaging.^[^ ^9^, ^10^, ^11^ ^]^

Hybrid bonding eliminates the need for solder bumps, allowing significantly finer interconnect pitches and higher input/output densities. This enables advanced integration strategies including 2.5D and 3D stacking,^[^ ^12^ ^]^ chiplet‐based architectures,^[^ ^13^, ^14^ ^]^ and fan‐out wafer‐level packaging.^[^ ^15^, ^16^, ^17^ ^]^ By removing solder‐related volume and materials, hybrid bonding improves vertical integration density while also reducing parasitic capacitance and inductance key advantages for enhancing signal integrity and power efficiency. These benefits make hybrid bonding a central enabling technology for future systems that demand miniaturized form factors and high‐speed, energy‐efficient data exchange.^[^ ^18^, ^19^, ^20^, ^21^, ^22^ ^]^

Despite its advantages, hybrid bonding, primarily based on Cu—Cu interconnection, generally requires bonding temperatures exceeding 400 °C under mechanical pressure to promote atomic diffusion and grain growth across the interface.^[^ ^10^, ^23^, ^24^, ^25^, ^26^ ^]^ It is known that surface activation methods such as plasma treatment and acid treatment can be employed to lower the bonding temperature; however, there remains a strong demand for research to develop more efficient low‐temperature strategies that ensure reliable Cu—Cu interconnection while maintaining compatibility with complementary metal‐oxide‐semiconductor and back‐end‐of‐line (BEOL) integration.^[^ ^27^, ^28^, ^29^, ^30^ ^]^ From the transistor standpoint, high bonding temperatures during front‐end‐of‐line processes can lead to thermal damage in device patterns; likewise, in BEOL semiconductor packaging, elevated thermal and mechanical loads significantly increase the thermal budget and induce interfacial stresses, causing reliability issues such as delamination, void formation, and crack propagation in adjacent dielectric layers such as SiO_2_. Furthermore, the mismatch in coefficients of thermal expansion between Cu and SiO_2_ dielectric layers can result in microcracking, voids, or delamination upon cooling, ultimately limiting compatibility in advanced packaging applications.^[^ ^31^, ^32^, ^33^ ^]^

To overcome these limitations, hybrid bonding processes should ideally be performed at lower temperatures to prevent thermal damage to transistors while still ensuring sufficient metal diffusion and strong adhesion between dielectric layers. One approach traditionally adopted in semiconductor packaging to reduce bonding temperatures is the use of solder bump interconnections composed of low‐melting‐point metals.^[^ ^34^, ^35^ ^]^ Accordingly, incorporating low‐temperature melting metals through methods such as electrodeposition, electroless metal deposition (ELD), or physical deposition can effectively reduce the overall processing temperature.

In this study, we present a hybrid bonding approach based on ELD onto Cu pads. This method enables selective metallization exclusively on exposed Cu pads with high chemical specificity, preventing unwanted deposition on adjacent SiO_2_ regions. Specifically, Au, Pt, and Sn were deposited on Cu pads as ultrathin interlayers (*ca.* 10–40 nm), significantly enhancing interfacial reactivity and metal diffusion during subsequent bonding processes. Additionally, a selective silylation treatment was applied to the SiO_2_ dielectric surfaces to improve adhesion at dielectric–dielectric interfaces. The entire bonding sequence, including a vacuum pre‐bonding step at 120 °C and final annealing at 250 °C, demonstrates a marked reduction in both thermal budget and process time compared to conventional hybrid bonding techniques. Even under these milder conditions, the bonded interfaces exhibit minimal void formation at Cu—Cu contacts and strong interfacial adhesion at SiO_2_—SiO_2_ boundaries. This strategy offers a scalable and efficient solution to overcome the inherent limitations of conventional interconnect technologies, positioning itself as a strong candidate for future ultradense semiconductor packaging platforms.

## 2 Results and Discussion

### 2.1 Low‐Temperature Hybrid Bonding Process for Advanced Packaging

**Figure 1:**
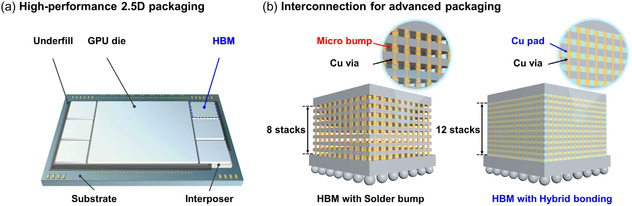
Schematic illustration of a) a high‐performance graphics processing unit (GPU) using 2.5D packaging with high‐bandwidth memory (HBM) integrated by an interposer and b) comparison of hybrid bonding interconnection technologies: solder bump‐based HBM with 8 memory stacks and hybrid bonding‐assisted HBM with 12 memory stacks. Insets highlight the differences between microbump connections and direct Cu—Cu bonding using Cu pads and vias.

**Figure** **1** illustrates the comparison of conventional solder bump interconnection and hybrid bonding. Solder bump interconnection is limited to about eight memory stacks due to its larger pitch and higher resistance, resulting in lower electrical and thermal performance. In contrast, hybrid bonding uses direct Cu—Cu bonding, enabling up to 12 stacks with better electrical, thermal, and mechanical performance. This makes hybrid bonding more suitable for advanced high‐performance semiconductor chips.^[^ ^36^, ^37^, ^38^, ^39^, ^40^, ^41^ ^]^

**Figure 2:**
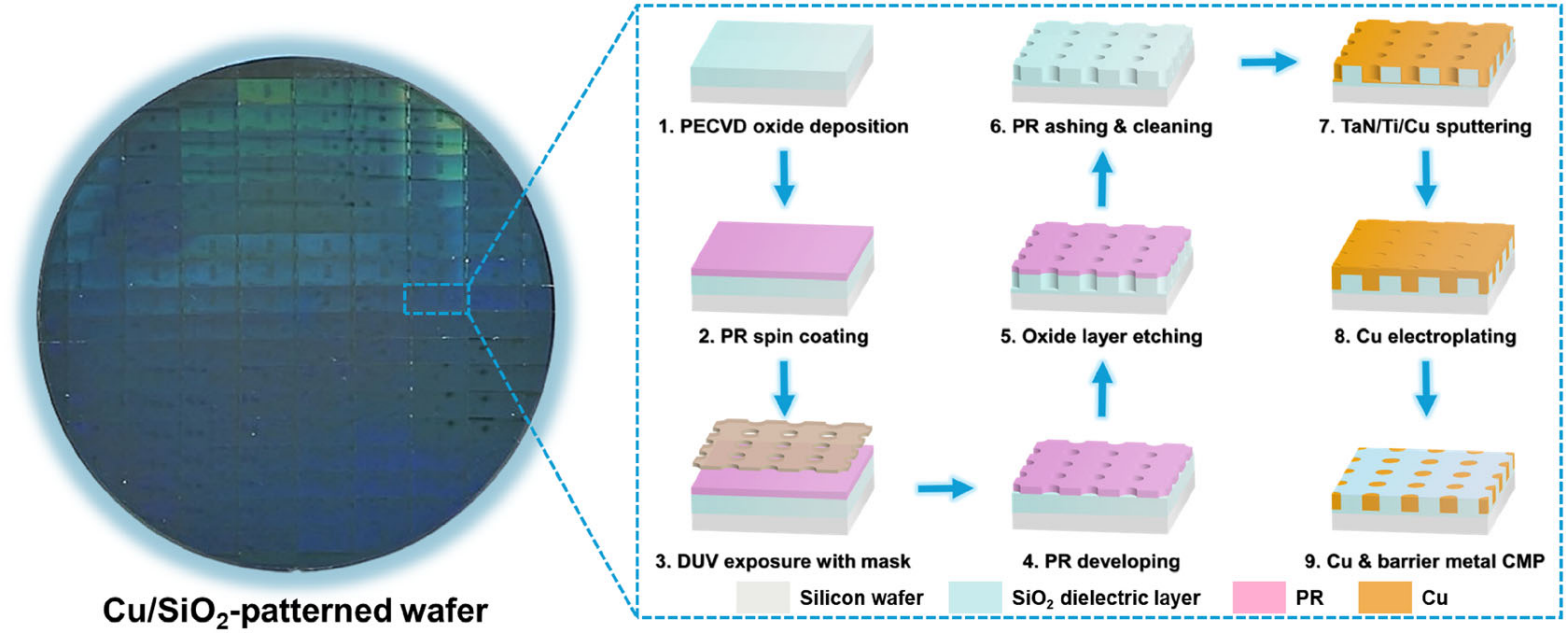
Digital photograph of the Cu/SiO_2_ wafer patterned for submicrometer‐pitch hybrid bonding, and schematic illustration of the sequential steps used to fabricate the Cu/SiO_2_ hybrid bonding chip, including lithographic patterning, etching, and metallization processes.

**Figure 3:**
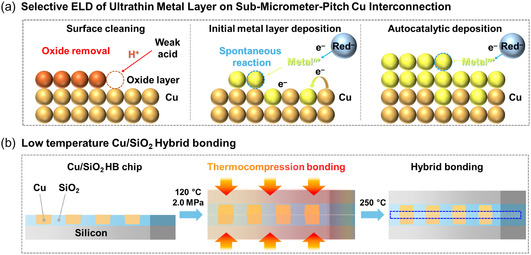
Schematic illustration of a) the ELD process and b) the hybrid bonding procedure. The ELD process involves three main steps: surface cleaning with a weak acid to remove the native oxide layer on Cu, spontaneous initial metal layer deposition via a redox reaction, and autocatalytic metal growth sustained by continuous electron transfer. The bonding process for sub‐micrometer‐pitch Cu/SiO_2_ hybrid bonding chips includes thermocompression bonding of two chips followed by formation of direct Cu—Cu and SiO_2_—SiO_2_ dielectric–dielectric interfaces, resulting in robust interconnection.

**Figure** **2** describes the manufacturing process for Cu/SiO_2_ hybrid bonding chip. Specifically, Cu/SiO_2_ wafers were prepared via a sequential process involving plasma‐enhanced chemical vapor deposition (PECVD), photolithography, damascene patterning, and chemical mechanical polishing (CMP). The processed wafers were subsequently diced into chips using a dicing method with controlled blade speed. The thicknesses of the electroplated Cu pads (*ca.* 750 nm) and SiO_2_ dielectric layer (*ca.* 850 nm) were carefully selected considering thermal expansion compatibility, thereby minimizing potential interfacial stresses arising from differences in thermal expansion coefficients during the hybrid bonding process. **Figure** **3** shows the schematic illustration of the metal ELD and hybrid bonding process. The ELD procedure consists of surface cleaning, initial metal layer deposition, and autocatalytic deposition (Figure 3a). The metals, including Au, Pt, and Sn, were selected as deposition metals because of their relatively low bonding temperatures compared with that of Cu, thereby enabling hybrid bonding under low‐temperature. Moreover, the successful implementation of these metals highlights the potential for heterogeneous metal integration, demonstrating the versatility and compatibility of the process with a wide range of materials. The thicknesses of the deposited metal layers were controlled by varying the immersion time. For the hybrid bonding process, two chips were precisely aligned and subjected to pre‐bonding (Figure 3b). During this step, dehydration and condensation reactions under vacuum condition facilitated the formation of hydrogen bonds between opposing SiO_2_ surfaces. Simultaneously, the applied compressive force minimized void formation and reduced the bonding gap. A subsequent annealing step was carried out in a nitrogen atmosphere to suppress Cu surface oxidation and further densify the bonding interface. The sequential low‐temperature bonding process involving vacuum pre‐bonding and post‐annealing resulted in high‐quality Cu/SiO_2_ hybrid bonding interconnections with strong structural integrity.

### 2.2 Surface Condition of Cu/SiO_2_ Hybrid Bonding Chip after Metal ELD and Silane Surface Modification

**Figure 4:**
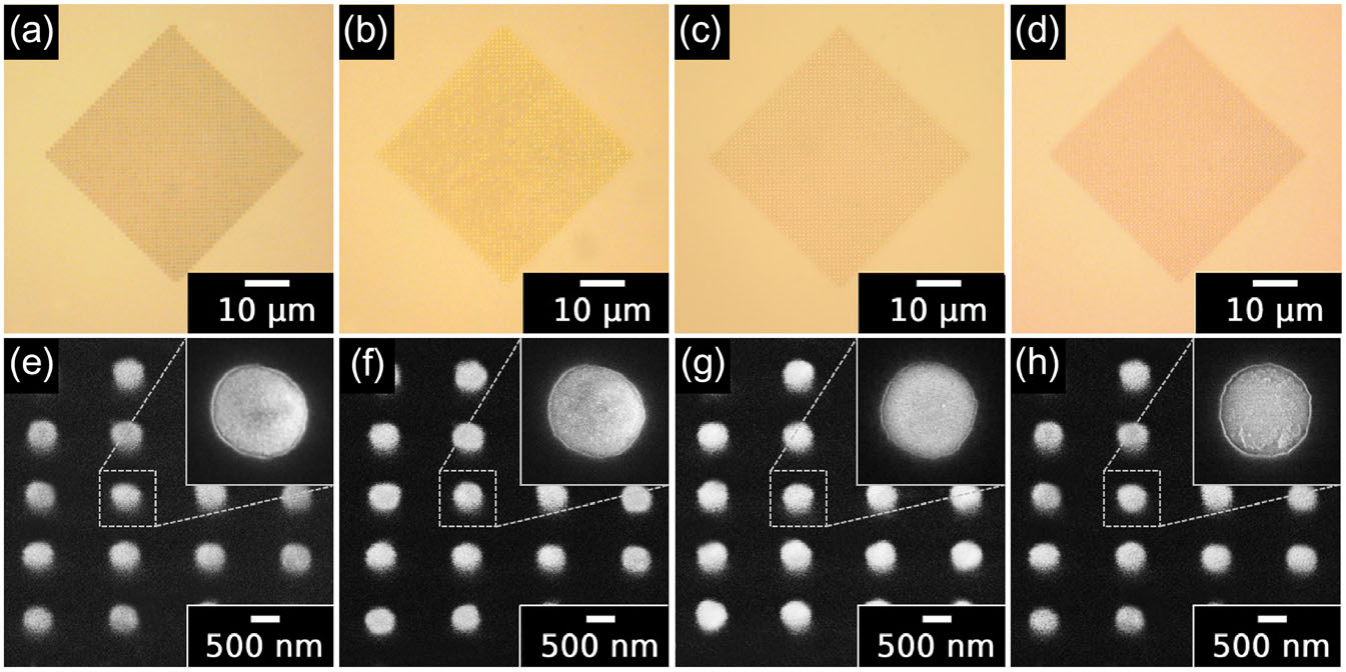
OM and FE‐SEM images of Cu/SiO_2_ hybrid bonding featuring uniformly patterned Cu pads (*ca.* 500 nm) before and after 20 s of ELD with various metals. OM images of a) pristine Cu/SiO_2_ chip, b) Au‐coated (ELD‐Au20@Cu/SiO_2_), c) Pt‐coated (ELD‐Pt20@Cu/SiO_2_), and d) Sn‐coated (ELD‐Sn20@Cu/SiO_2_) chips (scale bar: 10 μm). Corresponding FE‐SEM images showing the surface morphology of e) pristine, f) Au‐coated, g) Pt‐coated, and h) Sn‐coated chips (scale bar: 500 nm). All metal layers were uniformly deposited without distortion of the underlying sub‐micrometer patterns. Insets in (e–h) show magnified views of individual metal‐coated Cu pads.

The surface morphology of various metal‐deposited Cu/SiO_2_ hybrid bonding chips was systematically examined using optical microscopy (OM), as shown in **Figure** **4**a–d. The pristine Cu/SiO_2_ hybrid bonding chips exhibited uniformly patterned Cu pads with lateral dimensions of *ca.* 500 nm, characterized by clean, well‐defined square geometries (Figure 4a). Following metal deposition via the ELD process, the Cu pads displayed distinct and clearly observable color contrasts compared with the pristine chip, varying from reddish yellow to light green or pale yellow depending on the deposited metal (Figure 4b–d), thus confirming successful metal layer formation. Field‐emission scanning electron microscopy (FE‐SEM) images of pristine Cu/SiO_2_, ELD‐Au20@Cu/SiO_2_, ELD‐Pt20@Cu/SiO_2_, and ELD‐Sn20@Cu/SiO_2_ hybrid bonding chips are shown in Figure 4e–h. The pristine Cu/SiO_2_ hybrid bonding chip revealed smooth surfaces, sharply defined boundaries, and clearly patterned Cu pads *ca.* 500 nm in diameter. After the ELD process, significant morphological changes were observed on all metal‐coated chips. Specifically, ELD‐Au20@Cu/SiO_2_ and ELD‐Pt20@Cu/SiO_2_ exhibited continuous and uniform Au or Pt layers, characterized by clearly visible and densely distributed metal grains. These FE‐SEM observations explicitly confirmed uniform metal coverage without any evidence of microcracking or interfacial delamination. In contrast, ELD‐Sn20@Cu/SiO_2_ displayed distinctly textured surface morphology. Moreover, prolonged Sn deposition durations (exceeding 40 s) resulted in noticeable etching of the Cu pad surfaces. Overall, both OM and FE‐SEM analysis verified the successful surface modification of Cu pads through the fast processing metal ELD process (20 s), clearly demonstrating uniform surface alteration without structural degradation of the Cu pads at optimized deposition time.

The changes in surface morphology as a function of ELD duration are presented in Figure S1–S4, Supporting Information. Selective metal deposition onto the Cu pads was successfully achieved under all tested conditions; however, the resulting surface morphology varied significantly with deposition time. At shorter ELD durations (10 s), partial and discontinuous metal coverage was observed, indicating insufficient nucleation and limited growth of the deposited metal. With intermediate deposition times (30–40 s), surface roughening and slight discoloration were observed. In contrast, noticeable Cu etching and interfacial deterioration were observed at prolonged deposition durations (60 s), characterized by distinct etching and significant surface damage, particularly near the pad edges and centers. These findings clearly suggest that excessively long plating durations (60 s) lead to uncontrolled metal buildup and substantial Cu pad degradation, whereas shorter ELD durations (10–40 s) do not cause the etching or instability of the deposited metal layer.^[^ ^42^, ^43^, ^44^ ^]^

**Figure 5:**
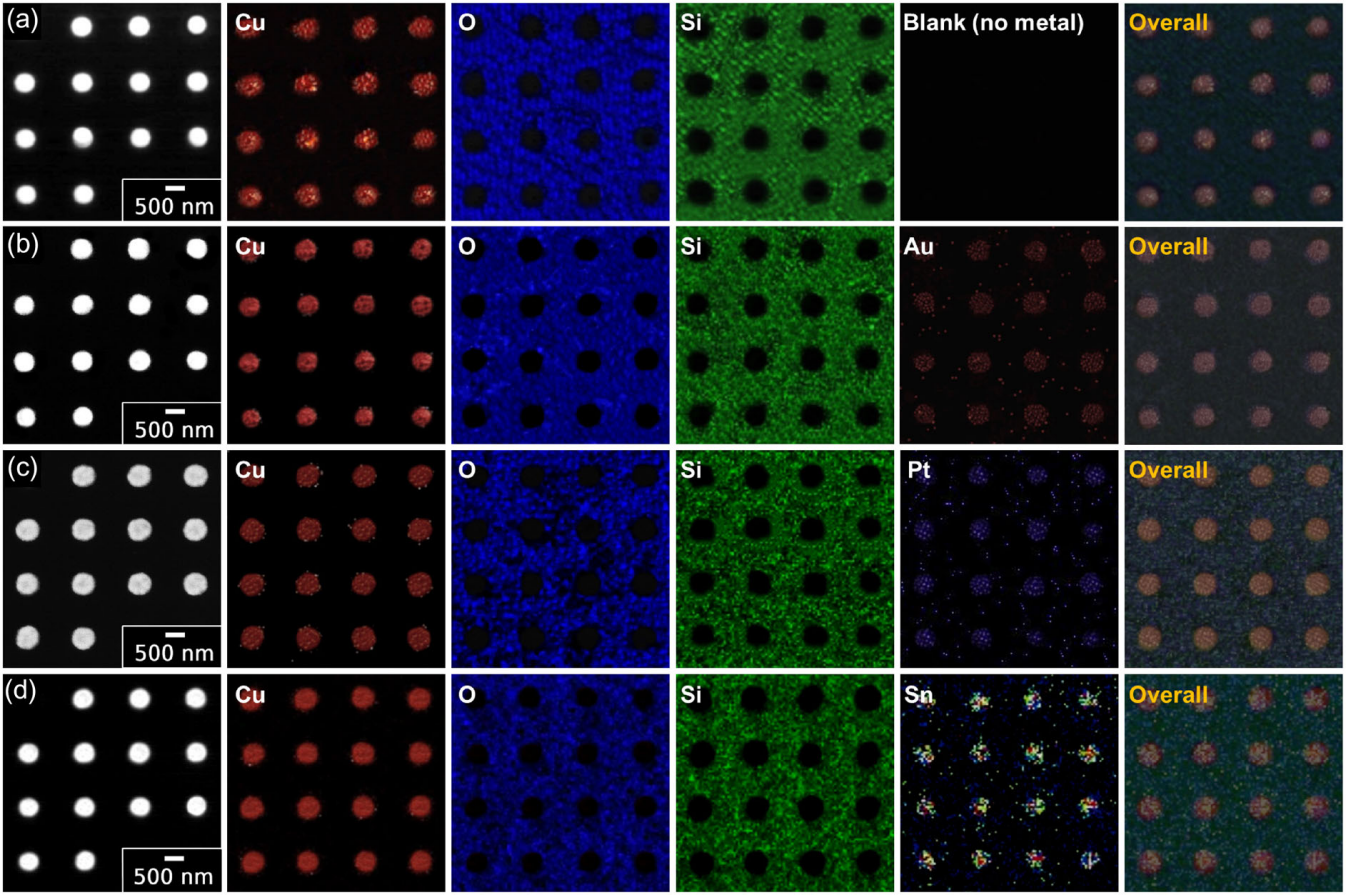
FE‐SEM and EDS elemental mapping images of Cu/SiO_2_ hybrid bonding before and after ELD with different metals. a) Pristine Cu/SiO_2_ chip, b) Au‐coated (ELD‐Au20@Cu/SiO_2_), c) Pt‐coated (ELD‐Pt20@Cu/SiO_2_), and d) Sn‐coated (ELD‐Sn20@Cu/SiO_2_) hybrid bonding chips (scale bar: 500 nm). EDS elemental mapping confirms selective metal deposition exclusively on the Cu pads, with no observable diffusion into the adjacent SiO_2_ dielectric regions. Detected elements: red = Cu, blue = O, green = Si, orange = Au, violet = Pt, and cyan = Sn.

In addition, green discoloration was observed at the outer edges of the Cu pads after Au and Pt deposition, which was attributed to residual chlorine species from the respective metal precursors. In addition, partial etching of the Cu surface was detected in ELD‐Au60@Cu/SiO_2_ and ELD‐Pt60@Cu/SiO_2_, likely due to chemical interactions between the Cu substrate and ELD bath constituents, including complexing agents, stabilizers, and reducing agents. These localized reactions accelerated Cu corrosion, resulting in surface deterioration. In addition, the successful removal of chlorine residues from the surfaces of ELD‐Au60@Cu/SiO_2_ and ELD‐Pt60@Cu/SiO_2_ hybrid bonding chips was confirmed by X‐ray photoelectron spectroscopy (XPS) and OM analysis (Figure S6–S8, Supporting Information). After cleaning by the IPA/sonication cleaning, the XPS spectra showed significantly reduced Cl2p peak intensity compared to as‐prepared samples, accompanied by clearly defined Au4f and Pt4f peaks. This result clearly demonstrates effective chlorine contamination removal, thus reducing the risk of surface corrosion and enhancing the overall bonding reliability.^[^ ^45^, ^46^, ^47^ ^]^ In contrast, ELD‐Sn60@Cu/SiO_2_ produced a relatively clean surface without visible residues; however, signs of Cu etching were still present. This phenomenon may be attributed to the sulfuric acid treatment employed during Cu activation, which enhances the surface reactivity and adhesion but simultaneously promotes Cu dissolution during ELD.^[^ ^48^, ^49^ ^]^ Due to current experimental limitations, comprehensive wafer‐level uniformity, bath stability, and reproducibility studies will be addressed in future work. The spatial distribution of the metal layers on the Cu pads was analyzed using energy‐dispersive X‐ray spectroscopy (EDS) elemental mapping.^[^ ^50^, ^51^, ^52^ ^]^ As shown in **Figure** **5**, all metals were selectively deposited onto the Cu pad regions within a fast processing time (20 s), displaying distinct elemental contrasts and highly localized distributions. The observed uniformity of metal coverage across Cu pads demonstrates the consistent control achieved through the ELD process. Importantly, no noticeable metal deposition was detected on the surrounding SiO_2_ dielectric layer areas, indicating selective deposition without lateral diffusion. The corresponding EDS spectra and quantified atomic compositions presented in Figure S5, Supporting Information, further validate the selective nature of the metal deposition.

**Figure 6:**
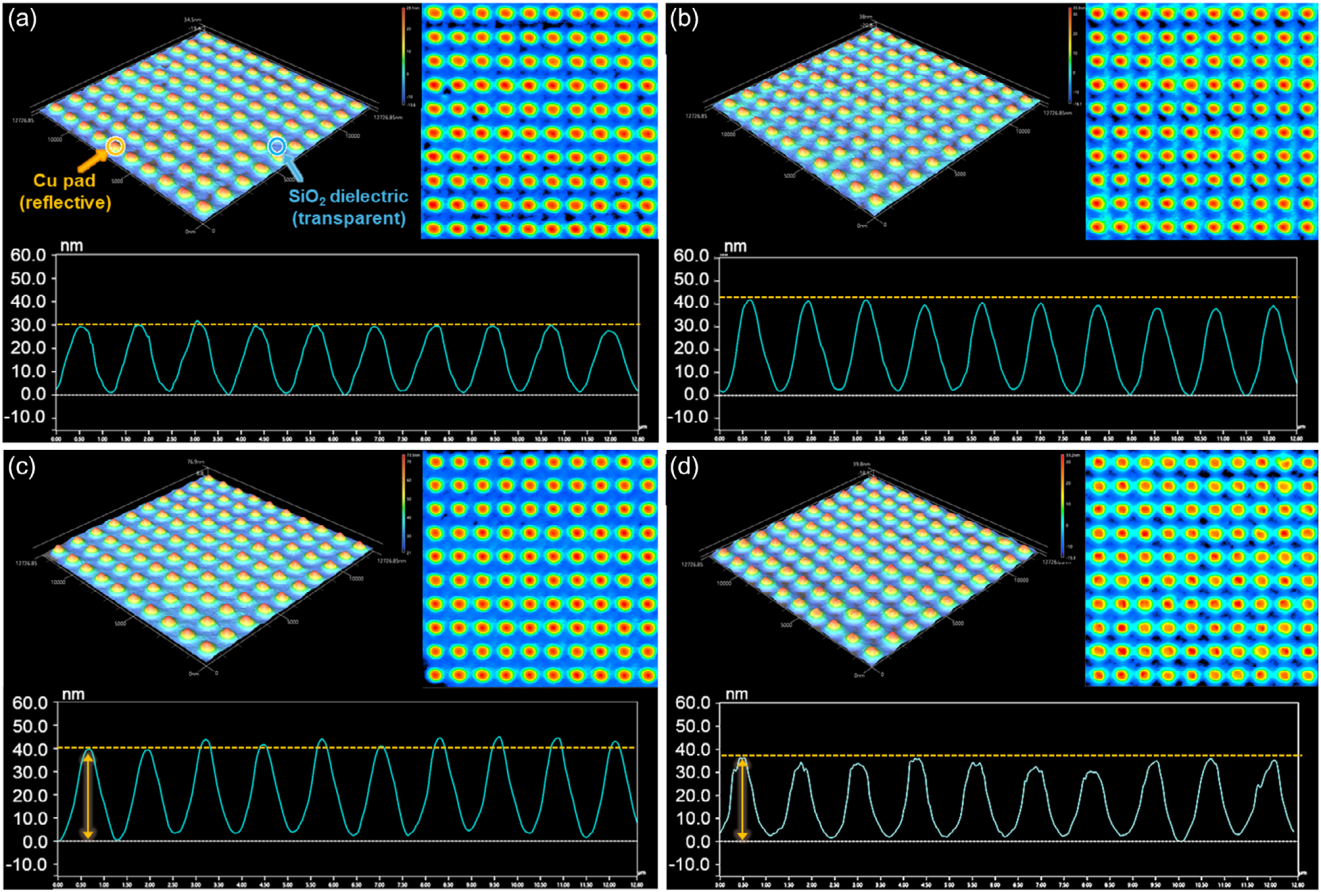
Optical profiler images of Cu/SiO_2_ hybrid bonding before and after 20 s of ELD with various metals. a) Pristine Cu/SiO_2_, b) Au‐coated (ELD‐Au20@Cu/SiO_2_), c) Pt‐coated (ELD‐Pt20@Cu/SiO_2_), and d) Sn‐coated (ELD‐Sn20@Cu/SiO_2_) hybrid bonding chip surfaces. Optically transparent SiO_2_ dielectric layers exist between the Cu pad or metal‐coated Cu pad. The profiles confirm uniform metal deposition with consistent step height and pattern fidelity across all treated hybrid bonding chips.

The surface roughness of the Cu/SiO_2_ hybrid bonding chips and the deposited metal thickness were evaluated using an optical profiler with multi‐mode calibration at high magnification. The pristine Cu/SiO_2_ hybrid bonding chip exhibited a low peak‐to‐valley height (*S*_z_), indicating a smooth and well‐defined Cu pad morphology. **Figure** **6**a–d shows the 3D surface topographies of the pristine Cu/SiO_2_, ELD‐Au20@Cu/SiO_2_, ELD‐Pt20@Cu/SiO_2_, and ELD‐Sn20@Cu/SiO_2_ hybrid bonding chips, respectively, along with their corresponding line roughness profiles derived from the 3D scans. The heights of the Au‐, Pt‐, and Sn‐deposited Cu pads were *ca.* 58.8, 59.0, and 57.9 nm, respectively. The ELD‐Au20@Cu/SiO_2_ hybrid bonding chip exhibits the lowest surface roughness, confirming the uniform and homogeneous nature of the Au layers. This smooth morphology may be attributed to the high deposition uniformity and strong interfacial affinity between Au and Cu, which promoted controlled metal growth. In contrast, ELD‐Sn20@Cu/SiO_2_ hybrid bonding chip exhibited the surface roughness, with an *S*_a_ value of 9.5 nm, suggesting a less uniform metal deposition. The non‐uniform metal deposition result was observed as a non‐consistent shape in the line profile graph. Figure S9, Supporting Information, shows the optical profiler images and surface roughness profiles of the ELD‐Au@Cu/SiO_2_, ELD‐Pt@Cu/SiO_2_, and ELD‐Sn@Cu/SiO_2_ hybrid bonding chips as functions of the deposition time (10, 30, and 40 s) (Supporting Information). The thickness and surface roughness of the metal layers increased with deposition time. The detailed surface roughness parameters, including the average surface roughness (*S*_a_), root‐mean‐square roughness (*S*_q_), and maximum peak‐to‐valley height (*S*_z_), are listed in **Table** **1** and S1, Supporting Information. The thicknesses of deposited metals (Au, Pt, and Sn) were precisely controlled within 10–40 nm, effectively minimizing surface roughness and preventing uncontrolled metal migration, thus ensuring stable, uniform interfaces and maintaining the integrity of fine‐pitch interconnection. Furthermore, the surface roughness of sub‐micrometer‐pitch Cu/SiO_2_ hybrid bonding chips was quantitatively evaluated at the nanoscale using atomic force microscopy (AFM). The pristine Cu/SiO_2_ hybrid bonding chip exhibited the lowest average roughness (*R*_a_) value of *ca.* 1.92 nm. After metal electroless deposition (ELD), slight increases in surface roughness were measured to be *R*_a_ = 2.45 nm (ELD‐Au20), *R*_a_ = 2.72 nm (ELD‐Pt20), and *R*_a_ = 3.34 nm (ELD‐Sn20) (Figure S10, Supporting Information). Importantly, these measured roughness values remain well below the recommended threshold (below 5 nm), ensuring robust interfacial bonding quality and precise alignment accuracy required at sub‐micrometer pitches.^[^ ^53^, ^54^ ^]^

**Table 1:** Surface roughness of pristine Cu/SiO_2_, ELD‐Au20@Cu/SiO_2_, ELD‐Pt20@Cu/SiO_2_, and ELD‐Sn20@Cu/SiO_2_ hybrid bonding chips measured using an optical profiler.

Metal thickness	*S*_a_ [nm]a)	*S*_q_ [nm]b)	*S*_z_ [nm]c)
Pristine	8.5	10.2	41.1
Au20	8.8	11.3	58.8
Pt20	10.4	12.2	59.0
Sn20	9.5	11.7	57.9

a) *S*_a_ = average surface roughness.

b) *S*_q_ = root‐mean‐square surface roughness.

c) *S*_z_ = maximum peak‐to‐valley heights of the surface.

To further increase adhesion, GPTMS silane was selectively coated onto the SiO_2_ dielectric layer using a facile immersion method. Various characterizations, including fourier transform infrared spectroscopy (FT‐IR) analysis, contact angle measurement, OM analysis, and cross‐sectional FE‐SEM analysis, were conducted to evaluate the successful modification of the SiO_2_ dielectric layer by GPTMS silane (Figure S11, Supporting Information). From the FT‐IR analysis, the GPTMS@Cu/SiO_2_ hybrid bonding chip exhibited characteristic peaks at 1100 cm^−1^ (Si—O—CH_3_ stretching),^[^ ^55^ ^]^ 1191 cm^−1^ (C—C epoxy ring), and 1465 cm^−1^ (C—C bonds), along with broad peaks at 2844 and 2938 cm^−1^ corresponding to –CH_3_ symmetric stretching (Figure S11a, Supporting Information).^[^ ^56^, ^57^ ^]^ The same peaks were also observed in commercial‐grade GPTMS, and the overall spectrum of GPTMS@Cu/SiO_2_ matched well with that of pure GPTMS, confirming the successful coating of GPTMS onto the SiO_2_ surface. Moreover, contact angle measurements were conducted on both pristine and GPTMS‐treated Cu/SiO_2_ hybrid bonding chips (Figure S11b, Supporting Information). After GPTMS treatment, the contact angle decreased from 69.7° to 41.1°, indicating a change in hydrophilicity due to silane coating.^[^ ^58^, ^59^ ^]^ The OM observation verified that no visible changes occurred on the Cu pads after the silane treatment, indicating that the silane was selectively coated onto the SiO_2_ dielectric layer. Additionally, the thickness of the silane coating was verified by cross‐sectional FE‐SEM analysis. The thickness of the GPTMS silane on the SiO_2_ layer was found to be *ca.* 22 nm using a silane concentration of 3.0 wt% (Figure S11c, Supporting Information). These characterizations verified that the GPTMS layer was successfully introduced onto the SiO_2_ dielectric surface without interfering with the Cu region.

### 2.3 Low‐Temperature Cu/SiO_2_ Hybrid Bonding Process

**Figure 7:**
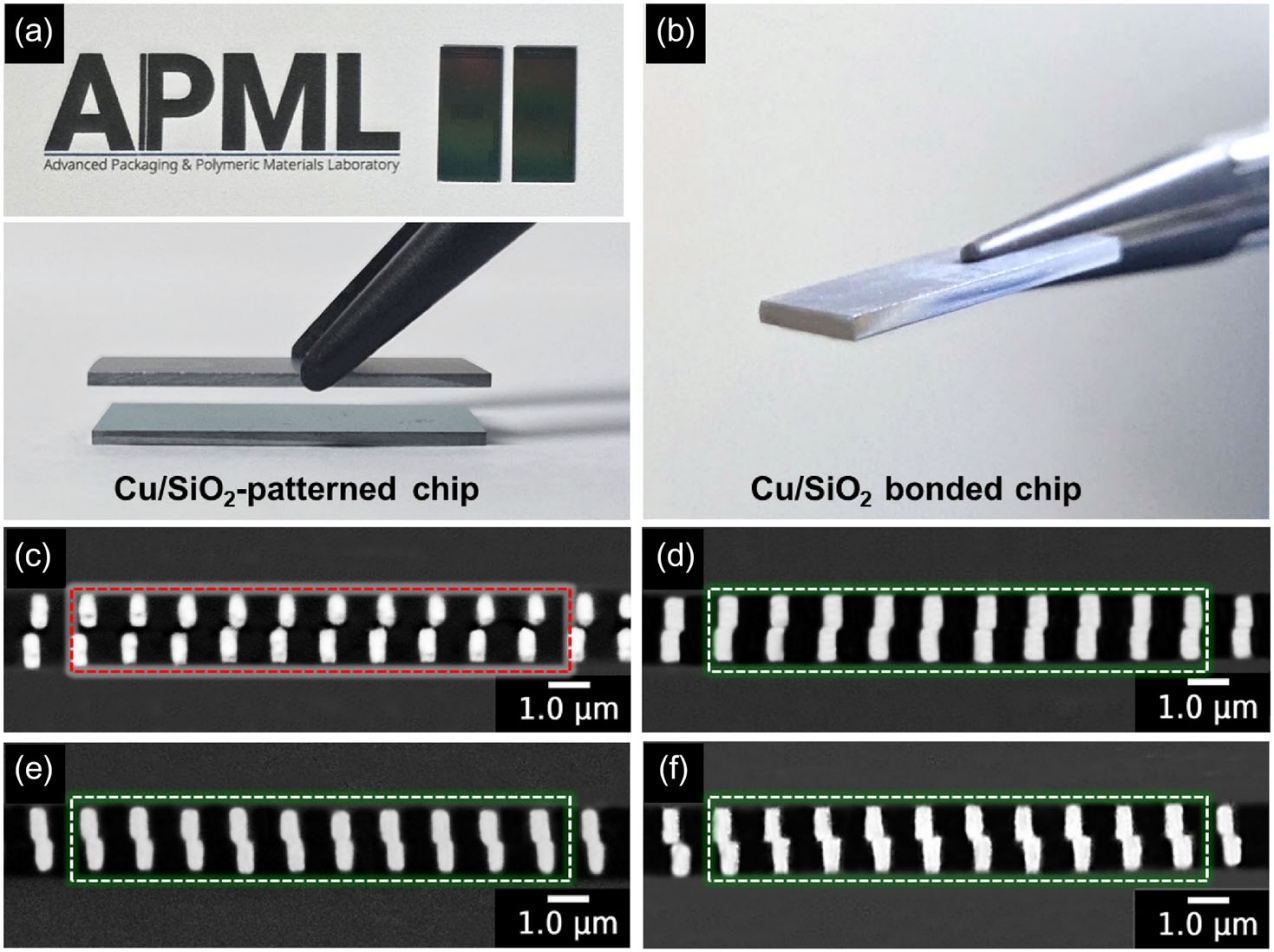
Digital photographs and cross‐sectional FE‐SEM images of Cu/SiO_2_ hybrid bonding chips. a) Top‐view photograph of Cu/SiO_2_‐patterned chips before bonding. b) Photograph of a successfully bonded Cu/SiO_2_ hybrid bonding chip. Cross‐sectional FE‐SEM images of the c) pristine Cu/SiO_2_, d) Au‐coated (ELD‐Au20@Cu/SiO_2_), e) Pt‐coated (ELD‐Pt20@Cu/SiO_2_), and f) Sn‐coated (ELD‐Sn20@Cu/SiO_2_) chip (scale bar: 1 μm). All bonded interfaces of samples except pristine Cu/SiO_2_ exhibit well‐aligned Cu and SiO_2_ regions with no visible interfacial voids or delamination.

To validate the structural integrity of the designed Cu/SiO_2_ hybrid bonding chip, thermal stability tests were performed on a single Cu/SiO_2_ hybrid bonding chip. Cross‐sectional FE‐SEM analysis confirmed the absence of delamination or void formation at the Cu/SiO_2_ interface before and after thermal treatment. Subsequently, two Cu/SiO_2_ chips were aligned face‐to‐face and bonded at 400 °C under a nitrogen atmosphere. The resulting cross‐sectional FE‐SEM images revealed no interfacial defects between Cu and SiO_2_, demonstrating the robustness of the selected dimensions under thermal stress (Figure S12, Supporting Information). Furthermore, hybrid bonding experiments conducted with these optimized dimensions at 250 °C under standard process conditions exhibited defect‐free interfaces, thereby validating the dimensional design and ensuring reliable structural stability at the targeted bonding temperature. Cross‐sectional FE‐SEM analysis was performed on samples with and without metal interlayers to evaluate the influence of metal ELD on the interfacial quality of submicrometer‐pitch Cu/SiO_2_ hybrid bonding chips (**Figure** **7**). All chips were prepared through a two‐step polishing process comprising mechanical polishing to eliminate surface irregularities and residual dielectric, followed by CMP to expose the Cu pads and ensure a flat, defect‐free cross‐section. A schematic illustration of the polishing mechanism and corresponding digital photograph of the polished surface are shown in Figure S13, Supporting Information. The hybrid bonding chip fabricated without metal ELD showed poor interfacial adhesion, as indicated by partial Cu pad detachment and the presence of unbonded regions at the Cu—Cu interface (Figure 7a,b). In contrast, all the metal ELD‐treated hybrid bonding chips exhibited successful Cu—Cu interconnections with significantly reduced interfacial gaps and improved structural coherence. The introduction of a metal layer on the Cu pads facilitated interfacial atomic diffusion during the subsequent annealing process, thereby enhancing the bonding integrity (Figure 7c–f). All metal‐deposited Cu/SiO_2_ hybrid bonding chips demonstrated a more robust interface, characterized by a well‐aligned Cu—Cu pad. These results indicate that the precisely controlled thickness of the intermediate metal layers directly influences the extent of atomic diffusion and interfacial connections achieved during bonding. Additionally, the void‐free SiO_2_—SiO_2_ bonding interface clearly demonstrates effective interfacial adhesion, attributable to the optimized GPTMS silane interlayer, which accommodates surface height variations and facilitates robust covalent bonding under thermal compression. Grazing‐incidence X‐ray diffraction (GI‐XRD) patterns were conducted to analyze the atomic‐scale interdiffusion behavior of pristine Cu/SiO_2_, ELD‐Au20@Cu/SiO_2_, ELD‐Pt20@Cu/SiO_2_, and ELD‐Sn20@Cu/SiO_2_ hybrid bonding chips (Figure S14, Supporting Information). The pristine Cu/SiO_2_ chip exhibited distinct diffraction peaks at 2θ values of ≈43.6°, 50.9°, and 74.8°, corresponding to the (111), (200), and (220) crystal planes of metallic Cu (JCPDS No. 04‐0836), confirming the absence of intermetallic phases at the bonding interface.^[^ ^60^, ^61^, ^62^ ^]^ In contrast, metal ELD‐treated samples showed clear peak shifts, additional diffraction peaks, and significant intensity variations. Specifically, for the ELD‐Sn20@Cu/SiO_2_ hybrid bonding chip, characteristic peaks associated with Cu_6_Sn_5_ were clearly observed at ≈30.1° (−113) and 52.8° (025), matched to JCPDS No. 45‐1488, along with peaks for Cu_3_Sn at ≈50.4° (200), matched to JCPDS No. 01‐1240.^[^ ^63^, ^64^, ^65^ ^]^ Interestingly, no clear evidence of IMC formation was observed for the Au‐ and Pt‐ELD‐treated hybrid bonding chips due to the intrinsic characteristics of Au and Pt. Although Au—Cu bonding at 250 °C has previously been demonstrated, stable Au—Cu intermetallic compounds (e.g., AuCu and AuCu_3_) typically form only at higher temperatures (above *ca.* 300–400 °C) with prolonged annealing times.^[^ ^66^ ^]^ Hence, the bonding between Au/Pt and Cu was dominated by direct metal‐to‐metal atomic diffusion across the interface, sufficient for robust bonding.

The long‐term reliability of the Cu/SiO_2_ hybrid bonding chip was evaluated by JEDEC Standard 22‐A103‐B at 180 °C (−0/+10 °C) for 280 h.^[^ ^67^, ^68^ ^]^ Post‐aging FE‐SEM analysis showed no gaps or voids at the Cu—Cu and SiO_2_—SiO_2_ interfaces (Figure S15, Supporting Information), confirming that metal ELD and hydrophilic silane treatment effectively preserved interfacial integrity under thermal stress.

Furthermore, cross‐sectional FE‐SEM analysis of the Cu/SiO_2_ hybrid bonding chips with varying ELD durations (Figure S16, Supporting Information) revealed a gradual increase in the metal layer thickness with increasing deposition time. However, excessive metal growth led to an expanded Cu—Cu bonding gap, which may hinder interface uniformity and compromise bonding reliability. These results emphasize the critical importance of controlling metal ELD parameters to achieve optimal metal coverage while maintaining defect‐free Cu—Cu metal bonding and SiO_2_—SiO_2_ dielectric layer interfaces. Figure S17a,b, Supporting Information, shows digital photographs of the Cu/SiO_2_ hybrid bonding chips prepared under optimal and excessive metal ELD conditions. A distinct visual difference between the optimally deposited and overdeposited metal layers is evident to the naked eye, highlighting the sensitivity of the bonding process to the deposition parameters. The corresponding cross‐sectional FE‐SEM images of the bonded interfaces are shown in Figure S17c–e, Supporting Information. Under optimal deposition conditions, the Cu—Cu interfaces exhibited narrow gaps, indicating close contact and successful interfacial diffusion. In contrast, chips subjected to excessive metal deposition exhibited irregular bonding gaps between the Cu pads. This degradation in bonding quality is attributed to the overgrowth of metal layers, which disrupts the surface planarity and hampers alignment during thermal compression. Such morphological inconsistencies can lead to localized stress concentrations and reduce the overall reliability of hybrid bonding interfaces.

**Figure 8:**
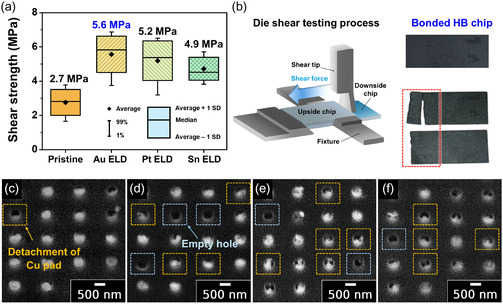
a) Bonding strength values of the hybrid bonded chips represented as box plots. Boxes represent mean ± 1 standard deviation (SD); central horizontal lines indicate the median values; whiskers indicate the data range; black diamond symbols (♦) indicate the mean values. b) Schematic illustration of the die shear test setup and corresponding digital photographs of the hybrid bonding chip before (top) and after (bottom) testing. Cross‐sectional FE‐SEM images of the fracture surfaces after die shear testing for c) pristine Cu/SiO_2_, d) Au‐coated (ELD‐Au20@Cu/SiO_2_), e) Pt‐coated (ELD‐Pt20@Cu/SiO_2_), and f) Sn‐coated (ELD‐Sn20@Cu/SiO_2_) hybrid bonding chips (scale bar: 500 nm). All images demonstrate the interfacial characteristics and failure modes associated with different metal treatments.

Lastly, the bonding performance of metal‐deposited Cu/SiO_2_ hybrid bonding chips was systematically evaluated by die shear testing, followed by surface analysis of the debonded interfaces using FE‐SEM (**Figure** **8**). The pristine Cu/SiO_2_ hybrid bonding chip bonded at 400 °C exhibited a shear strength of 2.7 MPa, with a peak load drop at 28.4 kg. In comparison, the ELD‐Au20@Cu/SiO_2_, ELD‐Pt20@Cu/SiO_2_, and ELD‐Sn20@Cu/SiO_2_ hybrid bonding chips with low‐temperature bonding at 250 °C showed significantly improved shear strengths of 5.6, 5.2, and 4.9 MPa, respectively (Figure 8a). Figure 8b shows the digital photograph of the sub‐micrometer‐pitch hybrid bonding chip before and after die shear tests, where a visible crack on the upper chip indicates mechanical failure under load.

Post‐shear surface analysis of the hybrid bonding chips were conducted using FE‐SEM to clearly identify fracture characteristics and Cu pad detachment behaviors (Figure 8c–f). For pristine Cu/SiO_2_ hybrid bonding chips, only a limited number of Cu pad detachments were observed, suggesting bonding failures primarily occurred at the Cu—Cu interface due to incomplete atomic diffusion at the low bonding temperature. In contrast, significantly greater Cu pad detachments and corresponding empty sites (vacancy of Cu pads) were clearly detected for ELD‐Au20@Cu/SiO_2_, ELD‐Pt20@Cu/SiO_2_, and ELD‐Sn20@Cu/SiO_2_ hybrid bonding chips. These observations indicate enhanced Cu—Cu atomic interdiffusion and robust metal–Cu interfacial adhesion achieved *via* the optimized metal ELD process. Consequently, the primary failure mode during die shear testing for ELD‐treated samples shifted predominantly to the SiO_2_—SiO_2_ interfaces. The observed differences relative to pristine samples can be directly attributed to variations in interfacial diffusion behaviors and adhesion characteristics of the deposited metals. Compared to current hybrid bonding processes, our proposed fast processing metal ELD method demonstrates clear advantages, achieving a lower bonding temperature of 250 °C, significantly finer alignment pitch (*ca.* 500 nm), and robust bonding strengths (*ca.* 5 MPa), thus highlighting its excellent suitability and high potential for advanced packaging.

## 3 Conclusion

This study presents a low‐temperature Cu/SiO_2_ hybrid bonding process enabled by rapid metal ELD for realizing sub‐micrometer‐pitch Cu interconnections in next‐generation semiconductor packaging. The bonding sequence involved three core steps: selective metal deposition via ELD, thermal pre‐bonding at 120 °C, and post‐annealing at 250 °C. Various metal precursors (Au, Pt, and Sn) were deposited onto the sub‐micrometer‐pitch Cu pads with precisely controlled thicknesses to evaluate their influence on the bonding behavior. Comprehensive surface and interfacial analysis, including OM, FE‐SEM, and optical profiler, indicated that all deposited metals yielded favorable results, characterized by minimal surface roughness, and continuous, void‐free Cu—Cu and SiO_2_—SiO_2_ interfaces. Among the metals, Au and Pt exhibited the highest interfacial integrity, which was attributed to its superior diffusivity, strong adhesion to Cu, and ability to promote atomic interdiffusion during annealing. From the die shear test, it was clear that the Au20@Cu/SiO_2_ hybrid bonding chips bonded at 250 °C exhibited a twofold increase in bonding strength compared to pristine Cu/SiO_2_ hybrid bonding chips bonded at 400 °C, highlighting the effective adhesion promotion enabled by ultrathin metal layers on Cu interconnections. Cross‐sectional FE‐SEM analysis confirmed that metal ELD‐assisted hybrid bonding process resulted in a narrow interfacial gap and enhanced the structural reliability. Additionally, excessive metal deposition was found to compromise the bonding uniformity by increasing the interfacial gaps and surface roughness, emphasizing the necessity of deposition duration control. The selective ELD‐assisted bonding method provides control over interface chemistry and ensures robust dielectric bonding through complementary surface silylation for achieving robust low‐temperature Cu/SiO_2_ hybrid bonding, particularly for sub‐micrometer‐pitch integration. This selective, low‐temperature metal coating strategy offers valuable insights for developing scalable, high‐density interconnects in next‐generation 3D packaging.

## 4 Experimental Section

4.1

4.1.1

##### Materials

Sodium hypophosphite monohydrate (NaH_2_PO_2_·H_2_O, 97.0%) was purchased from Junsei Chemical Co., Ltd. (Tokyo, Japan). Gold chloride trihydrate (HAuCl_4_·3H_2_O, 49.0% Au basis) was purchased from Merck KGaA (Darmstadt, Germany). Chloroplatinic acid hydrate (H_2_PtCl_6_·6H_2_O, 99.0%) was purchased from Kojima Chemicals Co., Ltd. (Tokyo, Japan). Absolute ethyl alcohol (99.9%), acetone (C_3_H_6_O, 99.0%), thiourea (CH_4_N_2_S, 98.0%), ethylenediamine (C_2_H_4_(NH_2_)_2_, 99.0%), tin(II) sulfate (SnSO_4_, 98.0%), L‐ascorbic acid (C_6_H_8_O_6_, 99.5%), and (3‐glycidyloxypropyl)trimethoxysilane (GPTMS, C_9_H_20_O_4_Si, 98.0%) were purchased from Samchun Chemical Co., Ltd. (Seoul, Korea). Isopropyl alcohol (IPA, (CH_3_)_2_CHOH, 99.5%) was purchased from Duksan Pure Chemicals Co., Ltd. (Ansan, Korea). All the chemicals were used without further purification.

##### Fabrication of Cu/SiO_2_‐Patterned Chips

Cu/SiO_2_‐patterned chips were fabricated using 8 inch silicon wafers. The surface of the Si wafer was cleaned using organic solvents to remove the contaminants and organic substances, and the SiO_2_ dielectric layer was deposited on the silicon wafer using PECVD method. After applying the UV 135G KrF‐positive photoresist (PR) by spin coating, the desired pattern was drawn using photolithography and etching using ASML PAS5500/300C/248nm step‐and‐scan system. After complete etching of SiO_2_ dielectric layer, the remained PR was removed by ashing. Subsequently, a TaN/Ti adhesion‐promoting layer was deposited onto a SiO_2_‐coated silicon wafer via sputtering, followed by Cu thin film which was deposited using an electroplating process. CMP was performed to remove the excess Cu to form the Cu pads with a lateral pitch of *ca.* 500 nm in the *x*–*y*‐direction and a vertical height of *≈*750 nm in the *z*‐direction. The thickness of the SiO_2_ dielectric layer was *ca.* 850 nm. Finally, blade dicing was performed on the wafer along the premarked lines using a diamond‐coated blade to obtain singular Cu/SiO_2_ hybrid bonding chips (length and width of 10 × 20 mm^2^).

##### Selective ELD of Metals (Au, Pt, and Sn) on Cu/SiO_2_ Hybrid Bonding Chip

The ELD procedure consists of surface cleaning, initial metal layer deposition, and autocatalytic deposition. Ultrasonication‐mediated surface cleaning was initially performed using an organic solvent to remove organic substances from the Cu/SiO_2_ chip. Selective metal deposition was then performed on a residue‐free Cu/SiO_2_ hybrid bonding chip using ELD. Specifically, a 10 mM metal stock solution was prepared using ultrapure water under a nitrogen atmosphere and stored in cold conditions to prevent unwanted reactions. The metal ELD solution was freshly prepared by diluting the stock solution and adding reducing and complexing agents under continuous stirring at 60 °C in a water bath for 1 h. Subsequently, the Cu/SiO_2_ chips were immersed in a metal ELD bath for controlled deposition times ranging from 10 to 40 s using a calibrated digital stopwatch with an automated timer‐controlled device, achieving the desired metal thickness while maintaining minimal surface roughness suitable for fine‐pitch hybrid bonding. Also, sample surfaces were cleaned with isopropyl alcohol (IPA) after the ELD to remove any excess deposits. The bath stability was maintained for 15 h of continuous use, attaining stable metal deposition performance. After deposition, the samples were rinsed with IPA to remove excess metal and stored under vacuum. Details of metal precursor, reducing agent, and complexing agent and their concentrations are listed in Table S2, Supporting Information. For comparison, excessive metal deposition was performed by extending the immersion time to 60 s. After metal deposition, the samples were cleaned by sonication in IPA for 5 min.

##### Low‐Temperature Two‐step Hybrid Bonding

A silylation step using hydrophilic GPTMS was conducted (pH ≤ 7, 60 min) after the metal ELD treatment, with the silane concentration adjusted from 1.0 to 7.0 wt%, to enhance adhesion of the SiO_2_ dielectric layers. The GPTMS‐treated Cu/SiO_2_ chips were placed in direct contact under ambient conditions. Thermal compression bonding was performed by heating the bottom plate to 120 °C while keeping the top plate at room temperature. The chips were maintained at 120 °C for 2 h under a compressive pressure of 2.0 MPa to facilitate the robust inorganic Si—O—Si network formation. Annealing was subsequently carried out at 250 °C for 2 h in a nitrogen atmosphere with a heating rate of 5 °C min^−1^. After annealing, the system was allowed to cool naturally to room temperature. Also, pristine Cu/SiO_2_ chips were bonded with same experimental conditions except annealing temperature has increased to 400 °C for die shear test comparison purpose.

##### Characterization

The metal deposition on the Cu/SiO_2_ chips was examined using FE‐SEM (SU‐8010, Hitachi) and OM (BH2‐UMA, Olympus). The distribution of the deposited metals on the Cu pads was examined using an EDS (EX‐250, Horiba) equipped to the FE‐SEM. The chemical bonding states and corresponding binding energies after chlorine removal were analyzed by XPS (K‐Alpha, Thermo Fisher Scientific). An optical profiler (VK‐X3050, Keyence) was used to measure the thickness of the metal deposition layer and change in the surface roughness of the Cu pad. Before conducting the surface roughness measurement, multi‐mode calibration of the system was conducted, including laser confocal scanning, focus variation, and white light interferometry, to ensure accurate acquisition of 3D height profiles and surface roughness across the patterned Cu pads. The surface roughness of the Cu pad at nanoscale resolution was analyzed using AFM (Multimode IVa, Veeco, USA) equipped with a J‐type scanner (100 × 100 × 5 μm^3^). The functional group and surface chemical state of the hybrid bonding chip were examined by FT‐IR (Nicolet iS10, Thermo Fisher Scientific). The hydrophilicity of the hybrid bonding chip was tested using a drop‐on‐demand generator, and the droplet size upon contact with the surface of hybrid bonding chip was captured by Navitar Zoom 7000 lens in a configuration at 1920 × 1080 resolution. The Cu/SiO_2_ hybrid bonding chips were subjected to a two‐step surface preparation process. Initial mechanical polishing was carried out to remove surface irregularities and dielectric residues, followed by CMP to expose the Cu pads and ensure a planar surface suitable for interface analysis. Mechanical polishing was conducted using One disc type polisher (GPM‐1000N, DreamTest), followed by CMP using an automatic precision lapping and polishing machine (UNIPOL‐1202, MTI Corporation) to expose the Cu pads and ensure a uniform surface for cross‐sectional FE‐SEM analysis. The alignment of Cu—Cu of the Cu/SiO_2_ hybrid bonded chips was analyzed using FE‐SEM (SU‐8010, Hitachi) after a two‐step polishing process. The crystallinity was analyzed using GI‐XRD (X’Pert PRO MRD, Malvern Panalytical) equipped with a Cu Kα X‐ray source (wavelength: 1.54056 Å; operating at 50 kV and 30 mA). The bonding strength and reliability of the hybrid bonding chips were evaluated by die shear tests (Dage 4000, Nordson) conducted at a shear height of 100 μm and a shear speed of 100 μm s^−1^. Chips with identical dimensions (10 × 10 mm) were bonded under identical conditions. The measured shear force (kgF) was converted to megapascals (MPa) by dividing the corresponding force in newtons by the bonding area (100 mm^2^). To ensure reliability, the tests were repeated five times under identical conditions, and the fracture surfaces of the hybrid bonding chips were examined using FE‐SEM.

## Supporting Information

Supporting Information is available from the Wiley Online Library or from the author.

## Conflict of Interest

The authors declare no conflict of interest.

## Supporting information

Supplementary Material

## Data Availability

The data that support the findings of this study are available from the corresponding author upon reasonable request.
