# Supplementary material for: A Selective Deposition Strategy of Ultrathin Metal Layer on Sub‐Micrometer‐Pitch Cu Interconnection for Low‐Temperature Hybrid Bonding
Source: Small Sci. 2025 Oct 11;6(2):e202500271. doi: 10.1002/smsc.202500271 (PMC12915207; doi:10.1002/smsc.202500271)
Supplement: Supplementary file 1 — Supplementary Material [file SMSC-6-e202500271-s001.pdf]

Supporting Information for

A Selective Deposition Strategy of Ultrathin Metal Layer on Sub-Micrometer-Pitch Cu Interconnection for Low Temperature Hybrid Bonding

Zambaga Otgonbayar[†1], *Jungchul Noh*[†2], *Seong-Ho Yoon*[3,4], *Gyu-Sik Park*[1,5], *Suk Jekal*[1,5], *Jiwon Kim*[1,5], *Jeong-Hwan Lee*[6,7,8], *Rino Choi*[6,7,8], *Jeonghun Kim*[9]*, *Myeongjin Kim*[10]*, *and Chang-Min Yoon*[1,5,8]*

[1] Z. Otgonbayar, G.-S. Park, S. Jekal, J. Kim, C.-M. Yoon

Department of Polymer Science and Engineering, Inha University, 100 Inha-ro, Michuhol-gu, Incheon 22212, Korea

E-mail: cmyoon4321@inha.ac.kr

[2] J. Noh

Department of Chemical Engineering, Hongik University, 94 Wausan-ro, Mapo-gu, Seoul 04066, Korea

[3,4] S.-H. Yoon

[3] Interdisciplinary Graduate School of Engineering Sciences, Kyushu University, 6-1 Kasugakoen, Kasuga, Fukuoka 816-8580, Japan

[4] Institute for Materials Chemistry and Engineering, Kyushu University, 6-1 Kasugakoen, Kasuga, Fukuoka 816-8580, Japan

[5] G.-S. Park, S. Jekal, J. Kim, C.-M. Yoon

Program in Environmental and Polymer Engineering, Inha University, 100 Inha-ro, Michuhol-gu, Incheon 22212, Korea

E-mail: cmyoon4321@inha.ac.kr

[6,7] J.-H. Lee, R. Choi

[6] Department of Materials Science and Engineering, Inha University, 100 Inha-ro, Michuhol-gu, Incheon 22212, Korea

[7] Program in Semiconductor Convergence, Inha University, 100 Inha-ro, Michuhol-gu, Incheon 22212, Korea

[8] J.-H. Lee, R. Choi, C.-M. Yoon

3D Convergence Center, Inha University, 100 Inha-ro, Michuhol-gu, Incheon, 22212, Korea

E-mail: cmyoon4321@inha.ac.kr

[9] J. Kim

Department of Chemical and Biomolecular Engineering, Yonsei University, 50 Yonsei-ro, Seodaemun-gu, Seoul 03722, Korea

E-mail: jhkim03@yonsei.ac.kr

[10] M. Kim

Department of Hydrogen & Renewable Energy, Kyungpook National University, 80 Daehak-ro, Bukgu, Daegu 41566, Korea

Email: myeongjinkim@knu.ac.kr

* Corresponding authors : Jeonghun Kim (E-mail: jhkim03@yonsei.ac.kr), Myeongjin Kim (E-mail: myeongjinkim@knu.ac.kr), Chang-Min Yoon (E-mail: cmyoon4321@inha.ac.kr)

Tel: +82-32-860-7487 (C.-M. Yoon)

List of Figures

- **Figure S1.** OM and FE-SEM images of (a, d) ELD-Au10@Cu/SiO_2, (b, e) ELD-Au30@Cu/SiO_2, and (c, f) ELD-Au40@Cu/SiO_2 hybrid bonding chips. The electroless deposition times were controlled at 10, 30, and 40 s, respectively.
- **Figure S2.** OM and FE-SEM images of (a, d) ELD-Pt10@Cu/SiO_2, (b, e) ELD-Pt30@Cu/SiO_2, and (c, f) ELD-Pt40@Cu/SiO_2 hybrid bonding chips. The electroless deposition times were controlled at 10, 30, and 40 s, respectively.
- **Figure S3.** OM and FE-SEM images of (a, d) ELD-Sn10@Cu/SiO_2, (b, e) ELD-Sn30@Cu/SiO_2, and (c, f) ELD-Sn40@Cu/SiO_2 hybrid bonding chips. The electroless deposition times were controlled at 10, 30, and 40 s, respectively.
- **Figure S4.** OM, FE-SEM, and high-magnified SEM images of (a–c) ELD-Au60@Cu/SiO_2, (d–f) ELD-Pt60@Cu/SiO_2, and (g–i) ELD-Sn60@Cu/SiO_2 hybrid bonding chips. The electroless deposition time was fixed at 60 s. Under extended deposition time, the Cu pads were overcoated by each metal.
- **Figure S5.** EDS spectra of a) pristine Cu/SiO_2, b) ELD-Au20@Cu/SiO_2, c) ELD-Pt20@Cu/SiO_2, and d) ELD-Sn20@Cu/SiO_2 hybrid bonding chips.
- **Figure S6.** Full XPS survey of the ELD-Au60@Cu/SiO_2 and ELD-Pt60@Cu/SiO_2 hybrid bonding chip before and after chlorine removal by IPA/sonication cleaning.
- **Figure S7.** High resolution XPS spectra of (a, b) Cu2p, c) Au4f, d) Pt4f, (e, f) Si2p, and (g, h) Cl2p for ELD-Au60@Cu/SiO_2 and ELD-Pt60@Cu/SiO_2 hybrid bonding chip before and after chlorine removal by IPA/sonication cleaning.
- **Figure S8.** OM image of the a) ELD-Au60@Cu/SiO_2, and b) ELD-Pt60@Cu/SiO_2 and corresponding OM images after chlorine removal for c) ELD-Au60@Cu/SiO_2, and d) ELD-Pt60@Cu/SiO_2, hybrid bonding chips.
- **Figure S9.** Optical profiler images of the a) ELD-Au10@Cu/SiO_2, b) ELD-Au30@Cu/SiO_2, c) ELD-Au40@Cu/SiO_2, d) ELD-Pt10@Cu/SiO_2, e) ELD-Pt30@Cu/SiO_2, f) ELD-Pt40@Cu/SiO_2, g) ELD-Sn10@Cu/SiO_2, h) ELD-Sn30@Cu/SiO_2, and i) ELD-Sn40@Cu/SiO_2 hybrid bonding chips [orange arrows indicate the uniform metal coating, red arrow = non-uniform metal coating].
- **Figure S10.** AFM surface topographies of the a) pristine Cu/SiO_2, b) ELD-Au20@Cu/SiO_2, c) ELD-Pt20@Cu/SiO_2, and d) ELD-Sn20@Cu/SiO_2 hybrid bonding chips.

- **Figure S11.** FT-IR spectra of the pristine Cu/SiO_2, GPTMS, and GPTMS@Cu/SiO_2 hybrid bonding chips. b) Contact angle and OM images of the pristine Cu/SiO_2 and GPTMS@Cu/SiO_2 hybrid bonding chips. c) Cross-sectional FE-SEM image of the silane-coated Cu/SiO_2 hybrid bonding chip, showing a silane layer thickness of 22 nm, and corresponding EDS elemental mapping (detected elements : C, O, Si).
- **Figure S12.** Digital photographs of a) a single Cu/SiO_2 chip, shown with cross-section FE-SEM images taken before (25 °C) and after thermal treatment (400 °C), and b) a Cu/SiO_2 bonded chip after thermal treatment (400 °C).
- **Figure S13.** Digital photograph of the UNIPOL-1202 automatic precision lapping and polishing machine and schematic illustration of the polishing mechanism for cross-section of hybrid bonding chips.
- **Figure S14.** GI-XRD spectra of the pristine, ELD-Au20@Cu/SiO_2, ELD-Pt20@Cu/SiO_2, ELD-Sn20@Cu/SiO_2 hybrid bonding chip measured at range of 20–80° 2θ degree.
- **Figure S15.** Cross-sectional FE-SEM images of the a) pristine Cu/SiO_2, b) ELD-Au20@Cu/SiO_2, c) ELD-Pt20@Cu/SiO_2, and d) ELD-Sn20@Cu/SiO_2 hybrid bonding chips after applied to the JEDEC Standard 22-A103-B condition (180 °C for 280 h).
- **Figure S16.** Cross-section FE-SEM images of (a–c) ELD-Au@Cu/SiO_2, (d–f) ELD-Pt@Cu/SiO_2, (g–i) ELD-Sn@Cu/SiO_2 hybrid bonding chips with thicknesses of *ca.* 10, 30, and 40 nm (left to right).
- **Figure S17.** Digital photographs of Cu/SiO_2 bonded chips prepared under a) optimal and b) excessive metal ELD conditions exhibiting seamless and widened interfacial gaps, respectively. Cross-section FE-SEM images of Cu/SiO_2 bonded chips with overcoated ELD layers: c) Au, d) Pt, and e) Sn.

List of Tables

- **Table S1**. Surface roughness values of various metal deposited Cu/SiO_2 hybrid bonding chips measured using an optical profiler.
- **Table S2**. Details of precursors and concentrations of metals, reducing agents, and complexing agents used in electroless deposition (ELD) process.

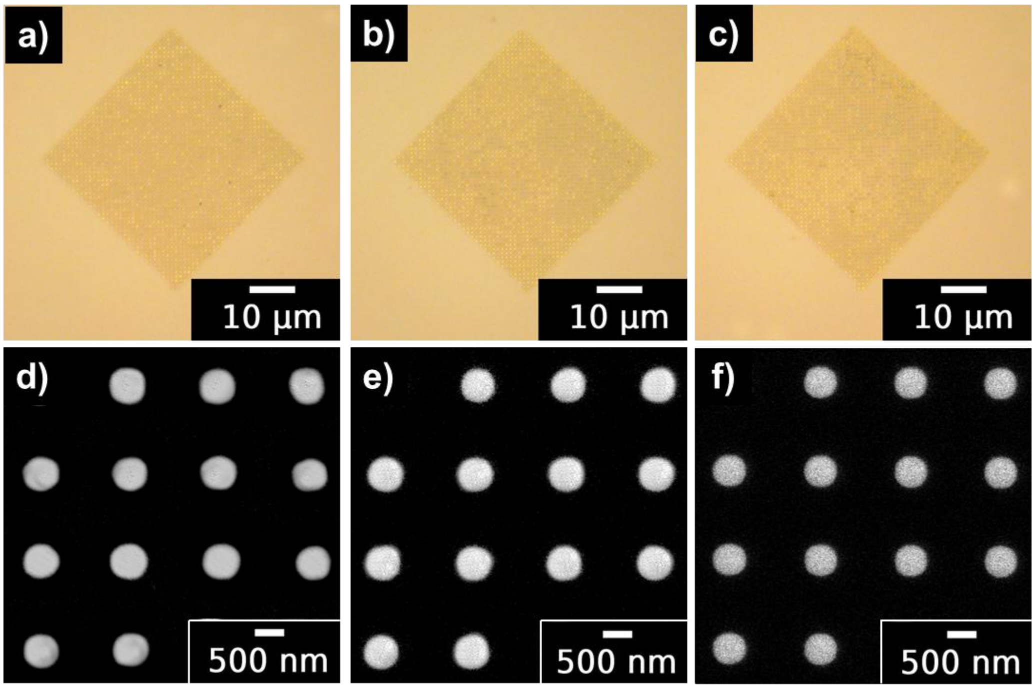

Figure S1. OM and FE-SEM images of (a, d) ELD-Au10@Cu/SiO_2, (b, e) ELD-Au30@Cu/SiO_2, and (c, f) ELD-Au40@Cu/SiO_2 hybrid bonding chips. The electroless deposition times were controlled at 10, 30, and 40 s, respectively.

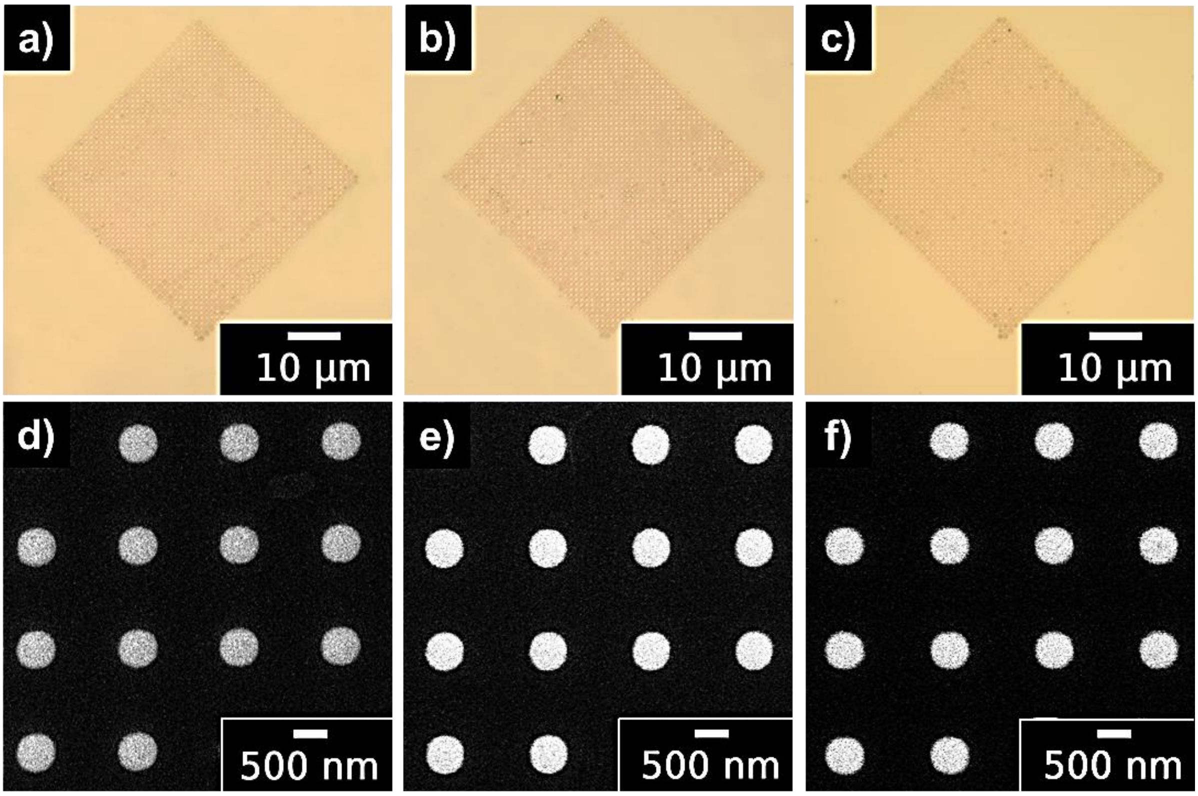

Figure S2. OM and FE-SEM images of (a, d) ELD-Pt10@Cu/SiO_2, (b, e) ELD-Pt30@Cu/SiO_2, and (c, f) ELD-Pt40@Cu/SiO_2 hybrid bonding chips. The electroless deposition times were controlled at 10, 30, and 40 s, respectively.

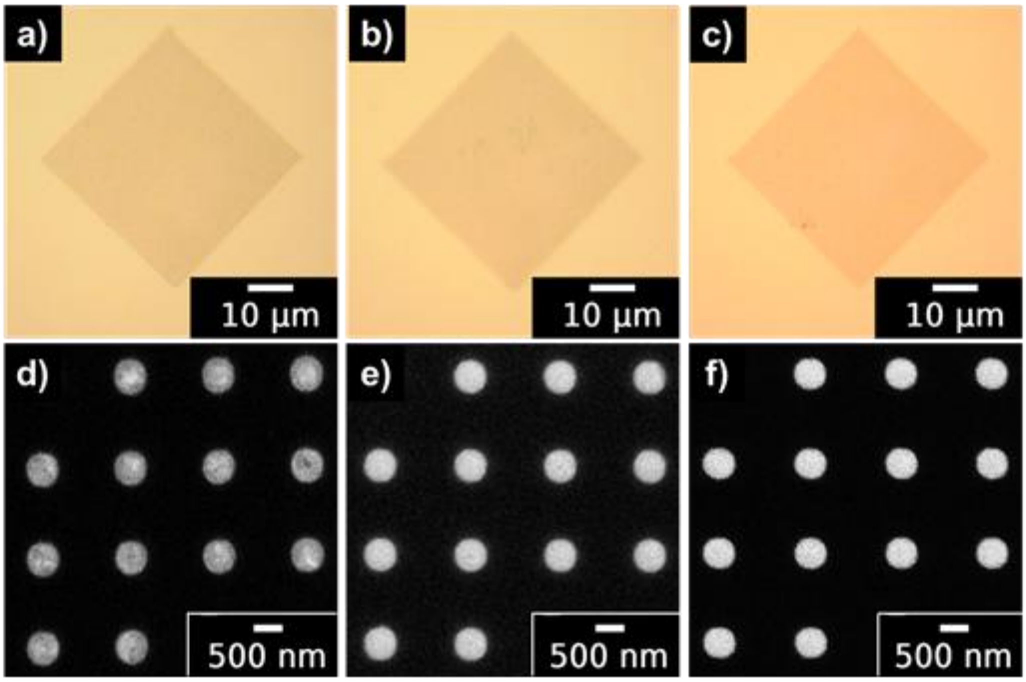

Figure S3. OM and FE-SEM images of (a, d) ELD-Sn10@Cu/SiO_2, (b, e) ELD-Sn30@Cu/SiO_2, and (c, f) ELD-Sn40@Cu/SiO_2 hybrid bonding chips. The electroless deposition times were controlled at 10, 30, and 40 s, respectively.

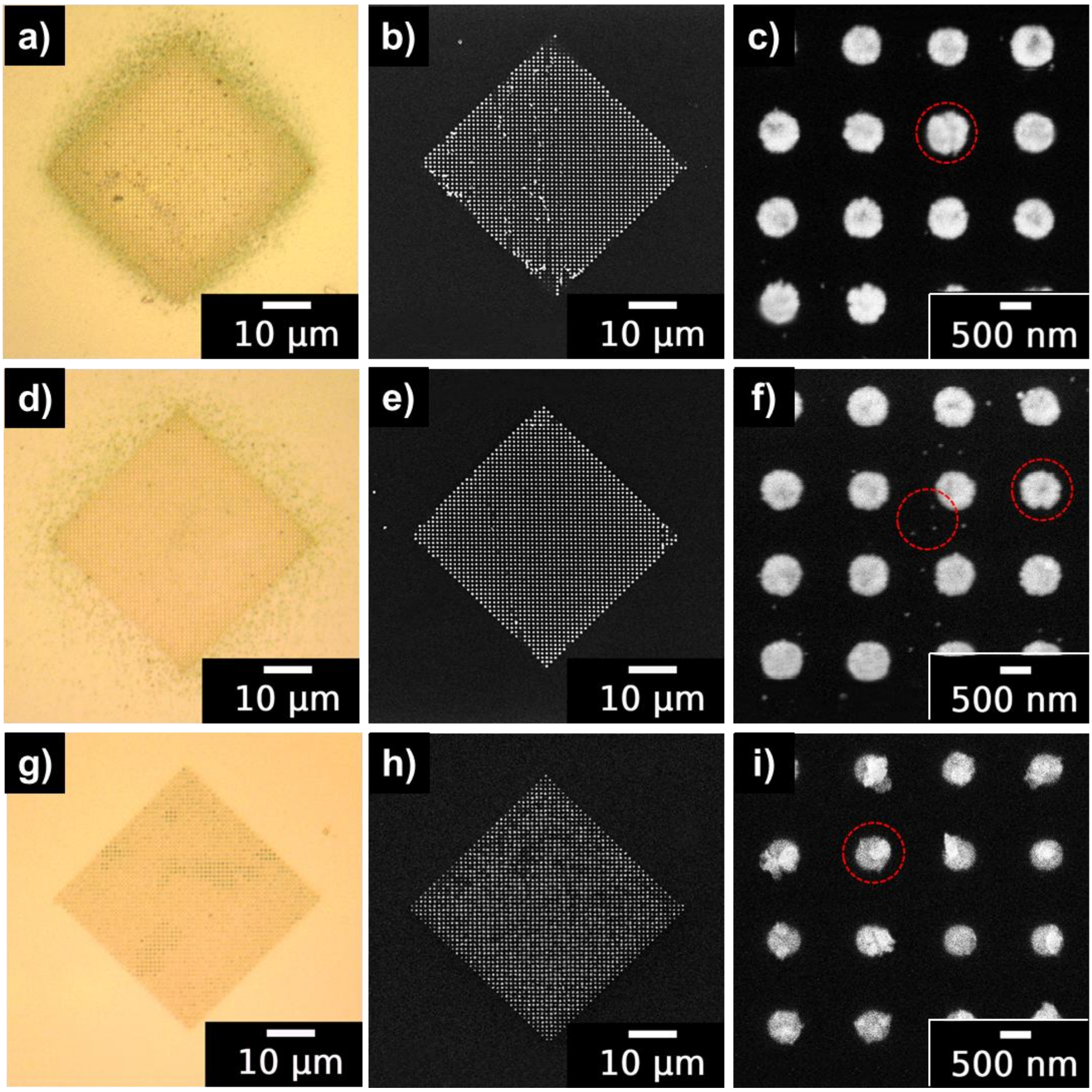

Figure S4. OM, FE-SEM, and high-magnified SEM images of (a–c) ELD-Au60@Cu/SiO_2, (d–f) ELD-Pt60@Cu/SiO_2, and (g–i) ELD-Sn60@Cu/SiO_2 hybrid bonding chips. The electroless deposition time was fixed at 60 s. Under extended deposition time, the Cu pads were overcoated by each metal.

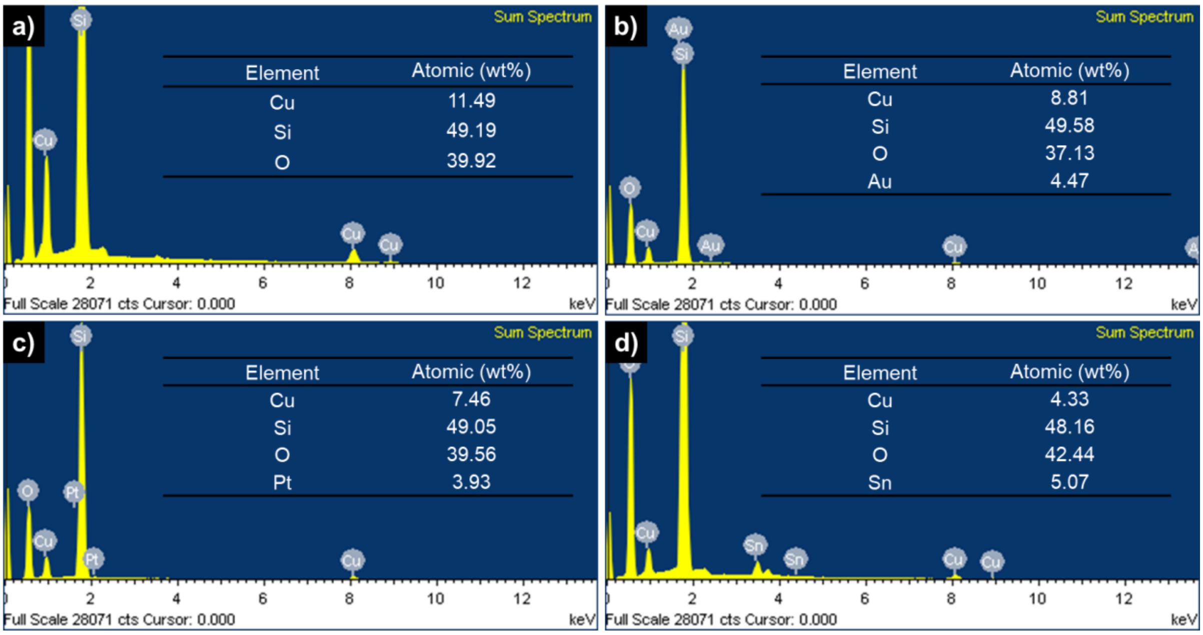

Figure S5. EDS spectra of a) pristine Cu/SiO_2, b) ELD-Au20@Cu/SiO_2, c) ELD-Pt20@Cu/SiO_2, and d) ELD-Sn20@Cu/SiO_2 hybrid bonding chips.

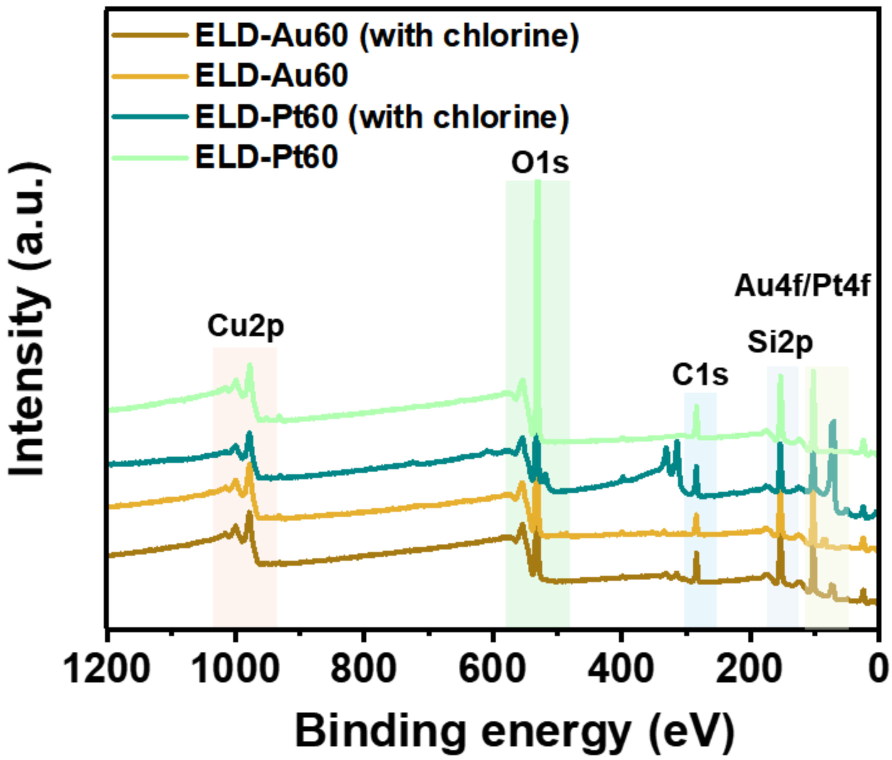

Figure S6. Full XPS survey of the ELD-Au60@Cu/SiO_2 and ELD-Pt60@Cu/SiO_2 hybrid bonding chip before and after chlorine removal by IPA/sonication cleaning.

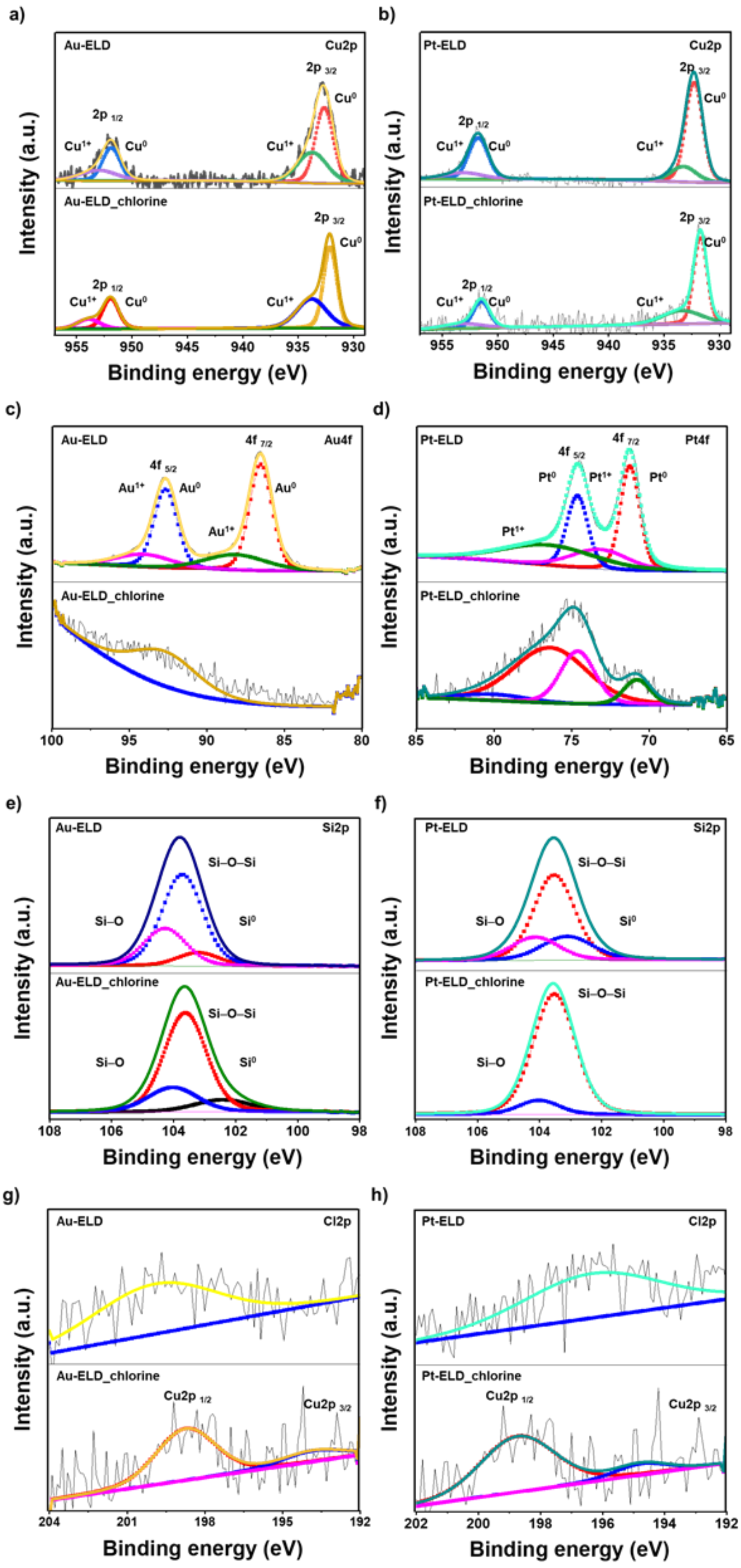

Figure S7. High resolution XPS spectra of (a, b) Cu2p, c) Au4f, d) Pt4f, (e, f) Si2p, and (g, h) Cl2p for ELD-Au60@Cu/SiO_2 and ELD-Pt60@Cu/SiO_2 hybrid bonding chip before and after chlorine removal by IPA/sonication cleaning.

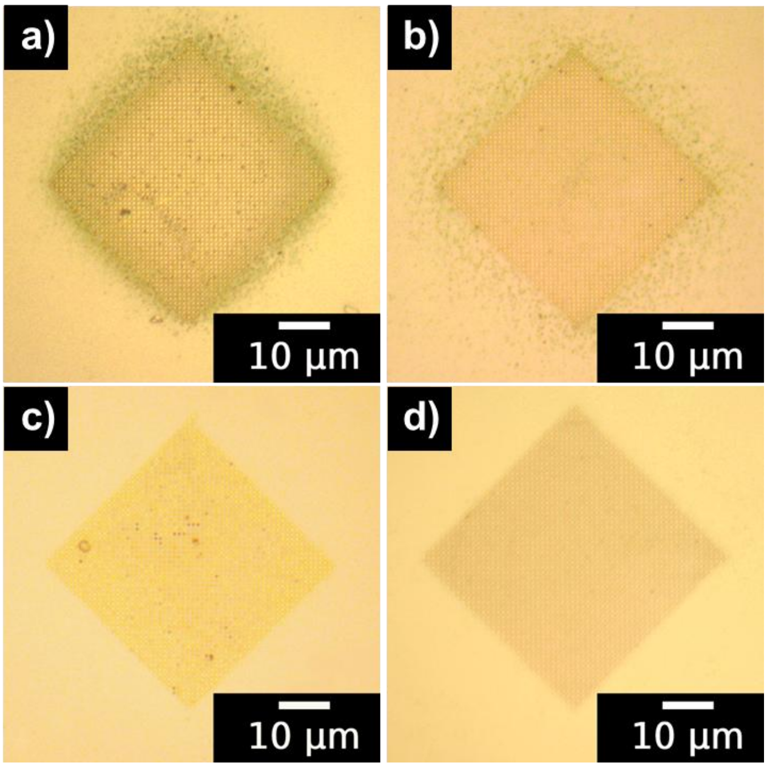

Figure S8. OM image of the a) ELD-Au60@Cu/SiO_2, and b) ELD-Pt60@Cu/SiO_2 and corresponding OM images after chlorine removal for c) ELD-Au60@Cu/SiO_2, and d) ELD-Pt60@Cu/SiO_2, hybrid bonding chips.

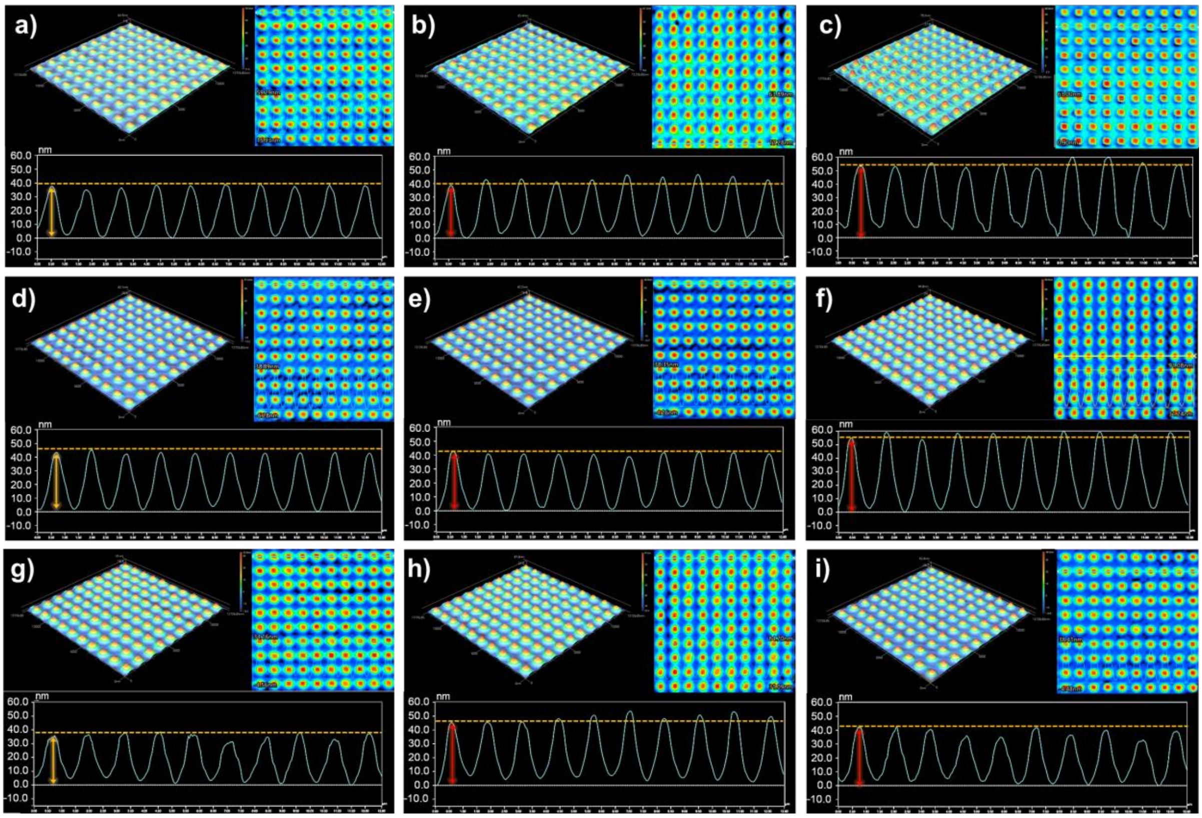

Figure S9. Optical profiler images of the a) ELD-Au10@Cu/SiO_2, b) ELD-Au30@Cu/SiO_2, c) ELD-Au40@Cu/SiO_2, d) ELD-Pt10@Cu/SiO_2, e) ELD-Pt30@Cu/SiO_2, f) ELD-Pt40@Cu/SiO_2, g) ELD-Sn10@Cu/SiO_2, h) ELD-Sn30@Cu/SiO_2, and i) ELD-Sn40@Cu/SiO_2 hybrid bonding chips [orange arrows indicate the uniform metal coating, red arrow = non-uniform metal coating].

Table S1. Surface roughness values of various metal deposited Cu/SiO_2 hybrid bonding chips measured using an optical profiler.

Metal thickness	S_a (nm)[a]	S_q (nm)[b]	S_z (nm)[c]
Au10	8.1	10.5	48.4
Au30	10.1	12.2	67.0
Au40	11.2	15.3	84.7
Pt10	9.5	11.6	49.9
Pt30	11.9	13.9	68.1
Pt40	12.8	15.1	74.2
Sn10	8.7	10.5	46.2
Sn30	9.6	11.8	65.4
Sn40	10.3	12.6	69.6

[a]S_a = average surface roughness.
[b]S_q = root-mean-square surface roughness.
[c]S_z = maximum peak-to-valley height of the surface.

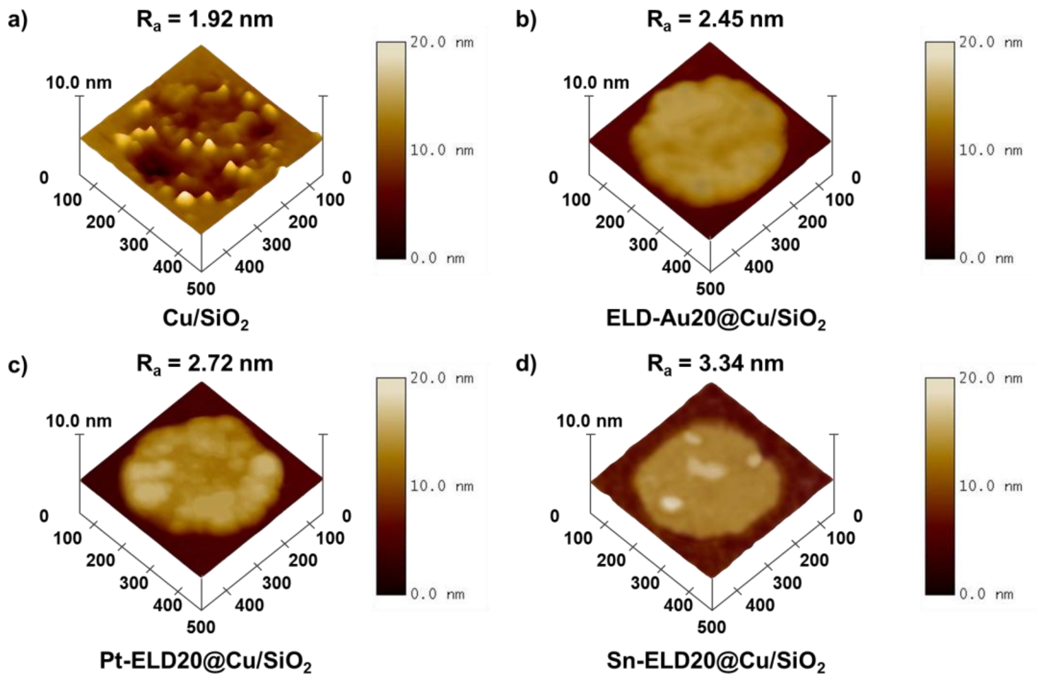

Figure S10. AFM surface topographies of the a) pristine Cu/SiO_2, b) ELD-Au20@Cu/SiO_2, c) ELD-Pt20@Cu/SiO_2, and d) ELD-Sn20@Cu/SiO_2 hybrid bonding chips.

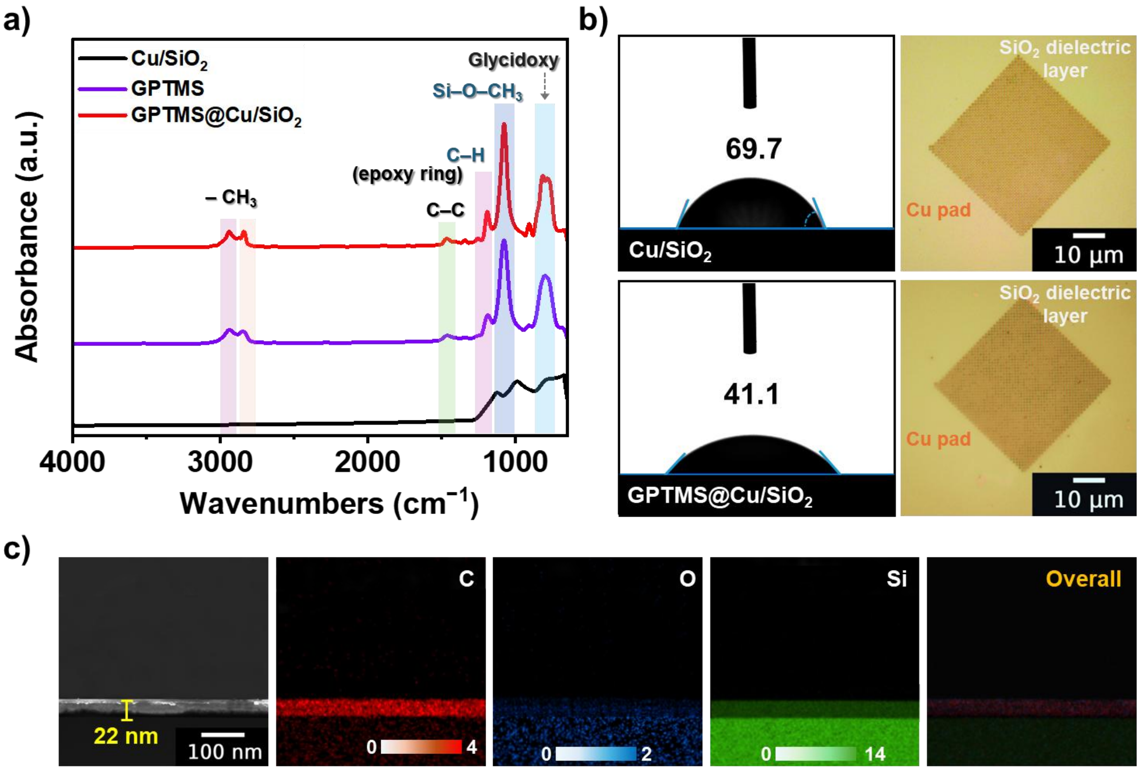

Figure S11. FT-IR spectra of the pristine Cu/SiO_2, GPTMS, and GPTMS@Cu/SiO_2 hybrid bonding chips. b) Contact angle and OM images of the pristine Cu/SiO_2 and GPTMS@Cu/SiO_2 hybrid bonding chips. c) Cross-sectional FE-SEM image of the silane-coated Cu/SiO_2 hybrid bonding chip, showing a silane layer thickness of 22 nm, and corresponding EDS elemental mapping images (detected elements: C, O, and Si).

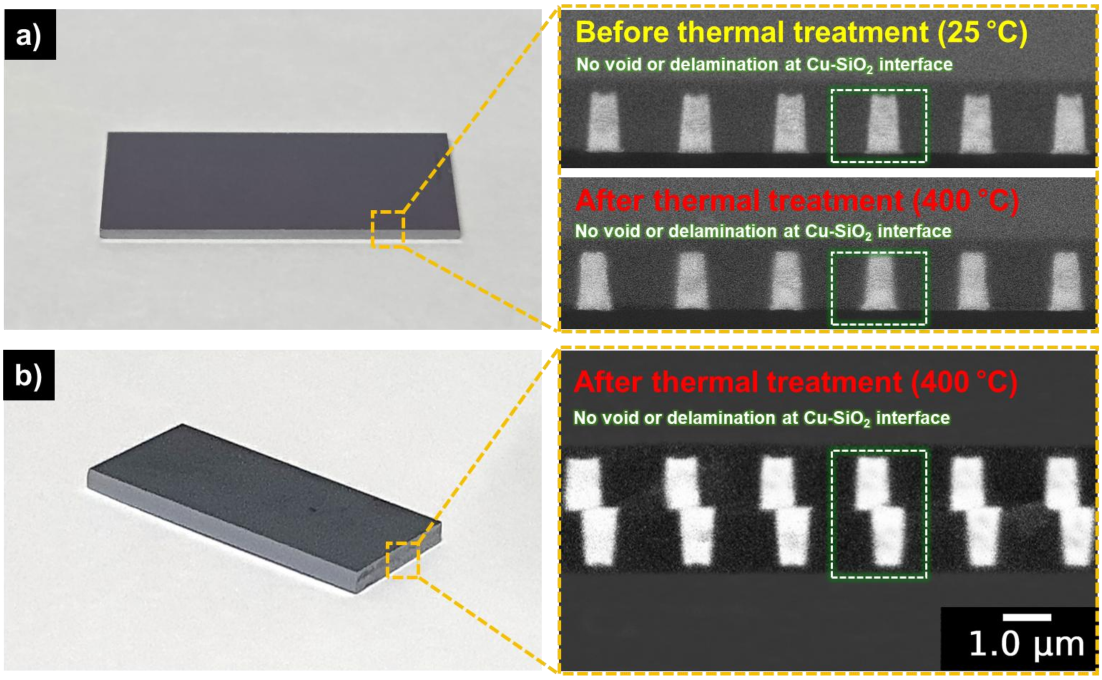

Figure S12. Digital photographs of a) a single Cu/SiO_2 chip, shown with cross-section FE-SEM images taken before (25 °C) and after thermal treatment (400 °C), and b) a Cu/SiO_2 bonded chip after thermal treatment (400 °C).

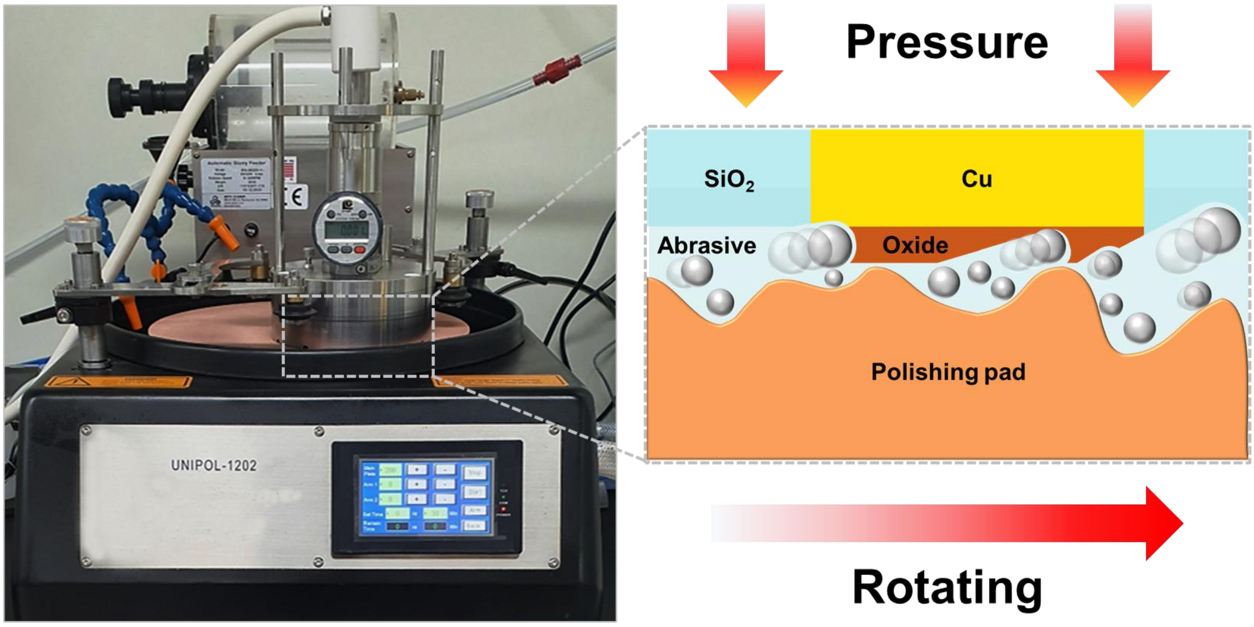

Figure S13. Digital photograph of the UNIPOL-1202 automatic precision lapping and polishing machine and schematic illustration of the polishing mechanism for cross-section of hybrid bonding chips.

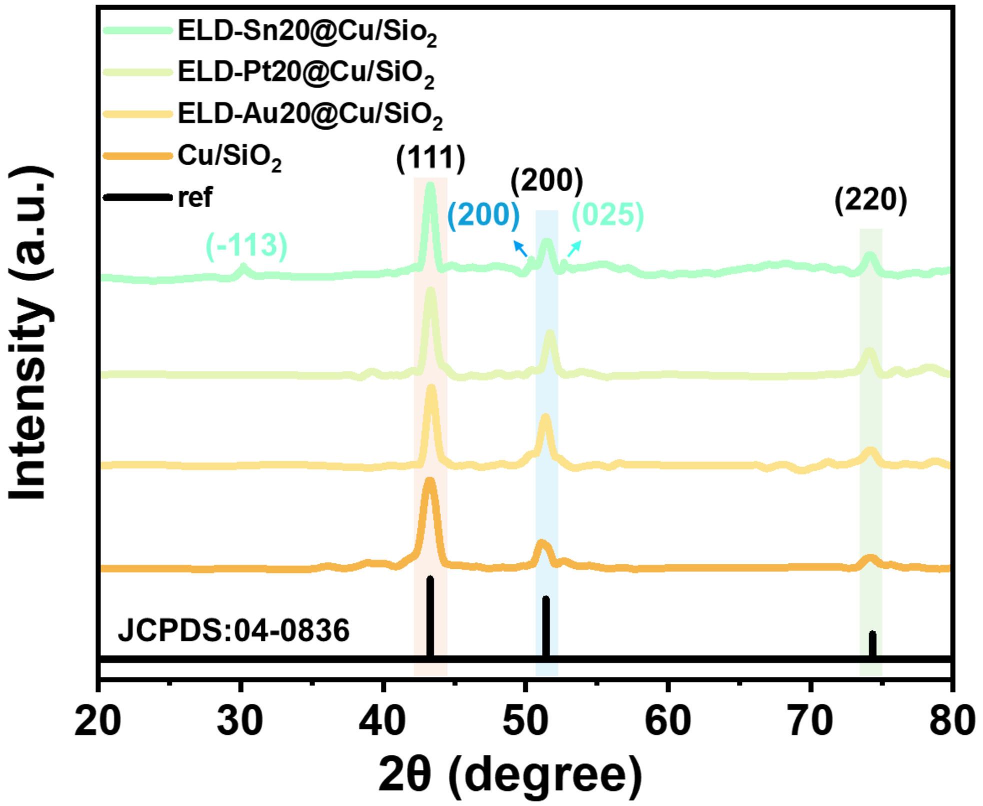

Figure S14. GI-XRD spectra of the pristine, ELD-Au20@Cu/SiO_2, ELD-Pt20@Cu/SiO_2, ELD-Sn20@Cu/SiO_2 hybrid bonding chip measured at range of 20–80° 2θ degree.

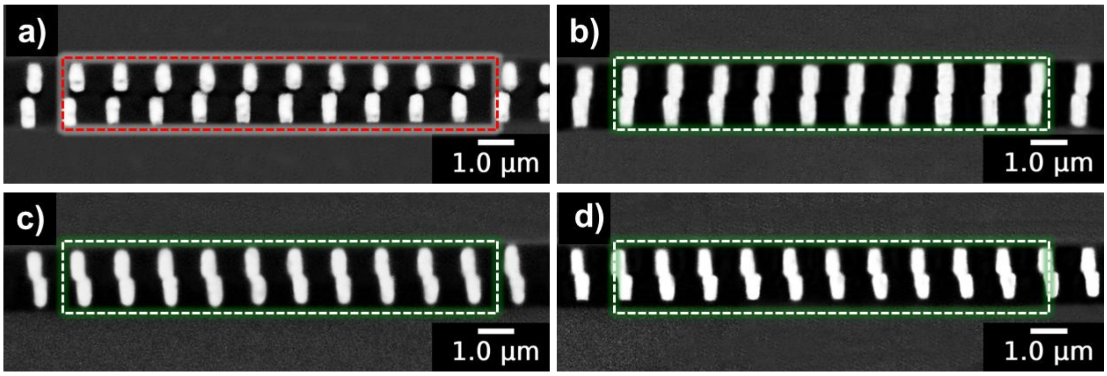

Figure S15. Cross-sectional FE-SEM images of the a) pristine Cu/SiO_2, b) ELD-Au20@Cu/SiO_2, c) ELD-Pt20@Cu/SiO_2, and d) ELD-Sn20@Cu/SiO_2 hybrid bonding chips after applied to the JEDEC Standard 22-A103-B condition (180 °C for 280 h).

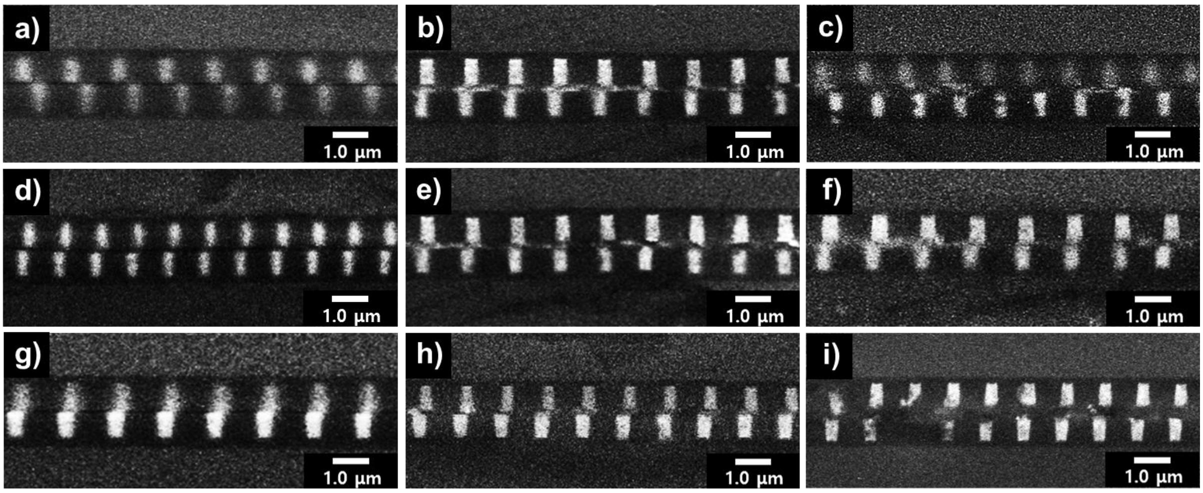

Figure S16. Cross-section FE-SEM images of (a–c) ELD-Au@Cu/SiO_2, (d–f) ELD-Pt@Cu/SiO_2, (g–i) ELD-Sn@Cu/SiO_2 hybrid bonding chips with thicknesses of *ca*. 10, 30, and 40 nm (left to right).

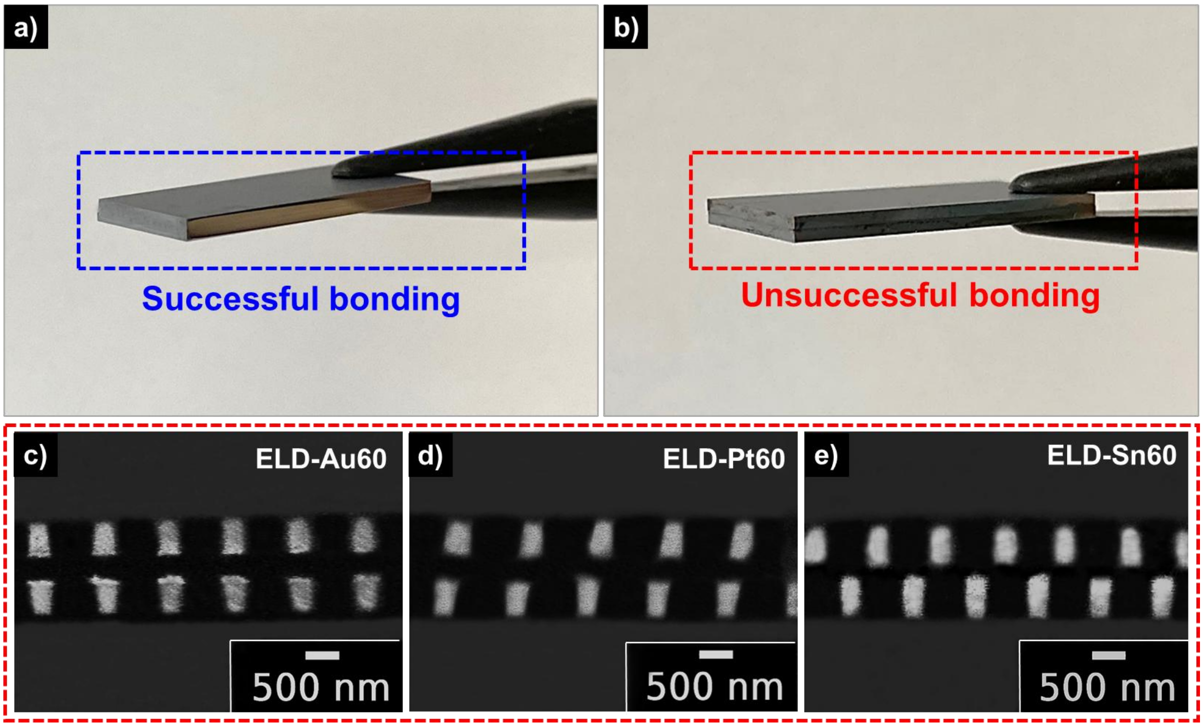

Figure S17. Digital photographs of Cu/SiO_2 bonded chips prepared under a) optimal and b) excessive metal ELD conditions exhibiting seamless and widened interfacial gaps, respectively. Cross-section FE-SEM images of Cu/SiO_2 bonded chips with overcoated ELD layers: c) Au, d) Pt, and e) Sn.

Table S2. Details of precursors and concentrations of metals, reducing agents, and complexing agents used in electroless deposition (ELD) process.[a]

Solution	Precursor	Reducing agent	Complexing agent	pH controller
Au-ELD	$HAuCl_4$ (0.4 mM)	NaH_2PO_2 (20 mM)	Ethylenediamine (20 mM)	KOH (target pH 10)
Pt-ELD	H_2PtCl_6 (0.15 mM)	Ascorbic acid (0.3 mM)	Sodium citrate (0.3 mM)	NaOH (target pH 7)
Sn-ELD	$SnSO_4$ (0.14 M)	NaH_2PO_2 (0.9 M)	Thiourea (1.05 M)	H_2SO_4 (target pH 1)

[a]All reagents were used without further purification.
